# *De Novo* Transcriptome Analysis to Identify Anthocyanin Biosynthesis Genes Responsible for Tissue-Specific Pigmentation in Zoysiagrass (*Zoysia japonica* Steud.)

**DOI:** 10.1371/journal.pone.0124497

**Published:** 2015-04-23

**Authors:** Jong Hwa Ahn, June-Sik Kim, Seungill Kim, Hye Yeon Soh, Hosub Shin, Hosung Jang, Ju Hyun Ryu, Ahyeong Kim, Kil-Young Yun, Shinje Kim, Ki Sun Kim, Doil Choi, Jin Hoe Huh

**Affiliations:** 1 Department of Plant Science, Seoul National University, Seoul, 151-921, Korea; 2 Research Institute of Agriculture and Life Sciences, Seoul National University, Seoul, 151-921, Korea; 3 Interdisciplinary Program in Agricultural Genomics, Seoul National University, Seoul, 151-921, Korea; 4 FnP Co., Ltd, Jeungpyeong, 368-811, Korea; McGill University, CANADA

## Abstract

Zoysiagrass (*Zoysia japonica* Steud.) is commonly found in temperate climate regions and widely used for lawns, in part, owing to its uniform green color. However, some zoysiagrass cultivars accumulate red to purple pigments in their spike and stolon tissues, thereby decreasing the aesthetic value. Here we analyzed the anthocyanin contents of two zoysiagrass cultivars ‘Anyang-jungji’ (AJ) and ‘Greenzoa’ (GZ) that produce spikes and stolons with purple and green colors, respectively, and revealed that cyanidin and petunidin were primarily accumulated in the pigmented tissues. In parallel, we performed a *de novo* transcriptome assembly and identified differentially expressed genes between the two cultivars. We found that two anthocyanin biosynthesis genes encoding anthocyanidin synthase (ANS) and dihydroflavonol 4-reductase (DFR) were preferentially upregulated in the purple AJ spike upon pigmentation. Both *ANS* and *DFR* genes were also highly expressed in other zoysiagrass cultivars with purple spikes and stolons, but their expression levels were significantly low in the cultivars with green tissues. We observed that recombinant ZjDFR1 and ZjANS1 proteins successfully catalyze the conversions of dihydroflavonols into leucoanthocyanidins and leucoanthocyanidins into anthocyanidins, respectively. These findings strongly suggest that upregulation of *ANS* and *DFR* is responsible for tissue-specific anthocyanin biosynthesis and differential pigmentation in zoysiagrass. The present study also demonstrates the feasibility of a *de novo* transcriptome analysis to identify the key genes associated with specific traits, even in the absence of reference genome information.

## Introduction

Zoysiagrass (*Zoysia japonica* Steud.) is a perennial creeping grass commonly found in Southeast and East Asia and Australia [1]. Zoysiagrass is widely used for lawns, mainly because of its quick spreading on the ground and fascinating green color under a broad range of environmental conditions [2]. Several zoysiagrass cultivars such as Meyer, Anyang-jungji, and Zenith are popular choices for warm-season lawn in Korea and other Northeast Asian countries.

Green appearance is one of the most commercial values of zoysiagrass. Most aerial parts of zoysiagrass are kept green under temperate climate, though creeping stolons and spike tissues often develop purple colors in some cultivars, which may compromise the uniform aesthetic value of green lawns. Therefore, development of new cultivars with less purple coloration is one of the breeding goals. For example, zoysiagrass cultivars Zenith and Millock have green spikes and stolons, whereas cultivars Meyer and Senock develop purple colors in the same tissues [3–6]. And most likely, the accumulation of anthocyanin pigments should result in purple coloration.

Despite its commercial values, only a limited number of molecular-based studies have been reported so far. Zoysiagrass is an allotetraploid (2n = 4x = 40) with a genome size of approximately 421 Mb [7, 8]. Even though several molecular linkage maps have been reported from *Z*. *japonica* and other *Zoysia* sp. [9–13], no comprehensive genetic studies have been done in zoysiagrass.

Anthocyanins, a group of phenolic compounds commonly found in many plant species, are responsible for red to purple colors in nature [14]. Anthocyanins are produced in various kinds of tissues in higher plants including leaves, stems, roots, flowers, and fruits. Anthocyanins play beneficial roles using their vivid colors, attracting pollinators and seed dispersers to flowers and fruits, and protecting cells from photooxidative damage in photosynthetic tissues by absorbing high-energy light [15, 16]. Anthocyanins are also well known for the antioxidant properties, alleviating oxidative stresses in plant tissues by scavenging free radicals, thus often used as a food additive for health benefit [17, 18].

The anthocyanin biosynthesis pathway is largely conserved among flowering plants. Flavonoid biosynthesis begins with the chalcone synthase (CHS) enzyme that utilizes a *p*-coumaroyl-CoA and three malonyl-CoAs to form a central flavonoid precursor tetrahydroxychalcone (naringenin chalcone), which is subsequently converted to flavanone naringenin by chalcone isomerase (CHI). Naringenin is either hydroxylated at the C-3 position within the central C-ring by flavanone-3-hydroxylase (F3H) to produce dihydroflavonol, or hydroxylated at the 3’ position of the B-ring by flavonoid 3’ hydroxylase (F3’H). Dihydroflavonols such as dihydromyricetin, dihydrokaempferol, and dihydroquercetin are subjected to diverse B-ring modifications by the action of flavonoid hydroxylases such as F3’H and flavonoid 3’, 5’-hydroxylase (F3’5’H), which primarily cause color differences among anthocyanin pigments. Consequently, the resulting dihydroflavonols serve as immediate precursors for the synthesis of flavonols and leucoanthocyanidins by flavonol synthase (FLS) and dihydroflavonol 4-reductase (DFR), respectively. Lecuoanthocyanidins are colorless but converted by anthocyanidin synthase (ANS) to colored anthocyanidins, to which a group of glycosyltransferases (GTs) then add diverse sugar moieties forming stable, water-soluble anthocyanin pigments. In addition to the hydroxylation at B-ring, the introduction of a methoxyl group also affects the color of anthocyanins.

To understand the genetic basis of purple pigmentation in zoysiagrass stolon and spikes, we analyzed the transcriptomes of two zoysiagrass cultivars displaying different colorations. *Z*. *japonica* ‘Anyang-jungji’ (AJ) shows purple coloration in stolon and spike tissues, while the other cultivar ‘Greenzoa’ (GZ) has green colors. By HPLC analysis, we demonstrate that anthocyanin accumulation is a main cause of different colorations of stolons and spikes between the two cultivars. To dissect the key components of anthocyanin pigmentation in zoysiagrass, a *de novo* transcriptome assembly was performed and we revealed that two anthocyanin biosynthesis genes *ZjDFR1* and *ZjANS1* were highly upregulated in purple-colored AJ spike and stolon tissues but hardly expressed in GZ. Finally, we performed *in vitro* assays with recombinant ZjDFR1 and ZjANS1 proteins showing that they sequentially catalyze the conversions of dihydroflavonols to anthocyanidins, forming leucoanthocyanidins as intermediates.

## Results

### Purple pigmentations of zoysiagrass are related to anthocyanin accumulation

**Fig 1:**
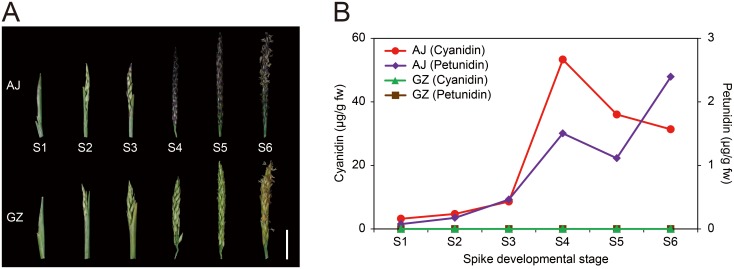
Differential pigment accumulation in AJ and GZ spikes. (A) Developmental stages of zoysiagrass spikes according to the size and the degree of pigmentation. ‘Anyang-Jungji’ (AJ) and ‘Greenzoa’ (GZ) spikes are categorized into six developmental stages (S1–S6) by size, color, and flower development. (B) Aglycone contents of acid-hydrolyzed anthocyanins in zoysiagrass spikes over developmental stages. The left *y*-axis indicates the amount of total cyanidin chloride and the right *y*-axis indicates the amount of petunidin chloride as determined by HPLC analysis. Fw, Fresh weight.

Two zoysiagrass cultivars ‘Anyang-jungji’ (AJ) and ‘Greenzoa’ (GZ) are phenotypically similar to each other except the color of spike and stolon (Fig 1A and S1 Fig). AJ spikes develop purple color during ripening, whereas GZ spikes keep green color until maturation. To analyze the temporal changes in purple pigmentation, we categorized developing spikes into six stages by their size, color, and floral organ structure (Fig 1A; Table 1). Developmental stages S1 and S2 are primarily defined by the size of spikes. Small spikes, or spikelets, at stage S1 are 20–25 mm long and hidden under the leaf sheath. Spikelets at stage S2 are 25–35 mm long and partially emerged from the leaf sheath. Stages S3 and S4 were categorized by the degree of coloration in AJ spikes and the equivalent sizes were applied to designate developmental stages of GZ spikelet. AJ spikes at S3 initiate coloration at the distal region, and 40–70% of spike body turns to purple at S4. Stigma and stamen of zoysiagrass emerge at different time points in order to prevent self-pollination [19], and this characteristic is applied to designate remaining stages S5 and S6. The spikes at stage S5 are fully-grown and the feathery stigma appears, whereas at stage S6, pendulous stamens emerge as the stigma starts to wither. In case of AJ, deep purple coloration of entire spike body is another characteristic at stages S5 and S6.

**Table 1:** Characteristics of AJ and GZ spikes during spike development.

Developmental stage	Average length (mm)		Color		Developmental characteristics
	AJ	GZ	AJ	GZ	
S1	21.3	20.9	Green	Green	Spikelet hidden inside the leaf sheath
S2	24.7	24.9	Green	Green	Partial spikelet emergence from the leaf sheath
S3	26.1	26.1	Partially purple (0–30%)	Green	Complete spike emergence and pigmentation initiation (AJ)
S4	30.4	29.8	Partially Purple (30–80%)	Green	Floret development and strong pigmentation in the lemma and palea
S5	36.2	35.9	Purple	Green	Emergence of feathery stigma from the floret
S6	36.8	36.5	Purple	Green	Emergence of pendulous stamens with stigma withering

Anthocyanins are red and purple pigments abundantly found in a broad range of plant species [14]. Anthocyanins are glycoside forms of anthocyanidins, and glycosylation is a major modification responsible for the solubilization and diversification of anthocyanin compounds [20]. Therefore, in an effort to identify major sugar-free anthocyanidin (aglycone) molecules that serve as immediate precursors for anthocyanin biosynthesis in zoysiagrass, we acid-hydrolyzed anthocyanins to remove sugar moieties and performed HPLC analysis [21, 22]. HPLC analysis revealed two major anthocyanin aglycones cyanidin and petunidin from AJ spikes (Fig 1B, S2 Fig). Even at green stages (S1–S2), relatively low levels of cyanidin and petunidin were detected from AJ spikes, and both accumulated during ripening (Fig 1B). In accordance with deep coloration, the spikes at stages S4 to S6 displayed high levels of anthocyanin accumulation (Fig 1B). Red-purple colored cyanidin was the most abundant, followed by blue-colored petunidin. By contrast, no apparent anthocyanin accumulation was observed in GZ spikes at any developmental stages. Luteolinidin, a kind of 3-deoxyanthocyanidin that is a 3-deoxy form of cyanidin, was not detected in AJ spikes (S3 Fig), suggesting that zoysiagrass has different profiles of flavonoid pigments compared to its close relatives such as *Sorghum bicolor*, in which yellow luteolinidin specifically accumulates as one of the major flavonoids [23]. These observations indicate that distinct coloration of AJ and GZ is determined by differential accumulation of anthocyanin pigments.

### *De novo* transcriptome analysis of zoysiagrass

As only limited genome information is available for zoysiagrass, we performed the Next Generation Sequencing (NGS)-based transcriptome analysis on mature spike tissues of AJ and GZ to identify candidate genes involved in the anthocyanin biosynthesis (stage S5 to S6; Fig 1A). From each of AJ and GZ spike tissues, approximately 44 Gbp of nucleotides were obtained and assembled into 28,561 and 28,984 mRNA contigs, respectively (Table 2 and S1 and S2 Tables). In both assemblies, the N50 size of assembled contigs was longer than 1,395 bp, with the longest contig being 15,359 bp long. The average contig lengths were 982.4 bp and 984.9 bp for AJ and GZ, respectively.

**Table 2:** Summary of *de novo* transcriptome assemblies of AJ and GZ.

	AJ	GZ
Total length (bp)	28,057,682	28,547,216
Number of contigs	28,561	28,984
Average length (bp)	982.4	984.9
Median length (bp)	721	715
Maximum length (bp)	15,205	15,359
Minimum length (bp)	200	200
N50 length (bp)	1,395	1,415
N80 length (bp)	699	700
GC content (%)	44	43

**Fig 2:**
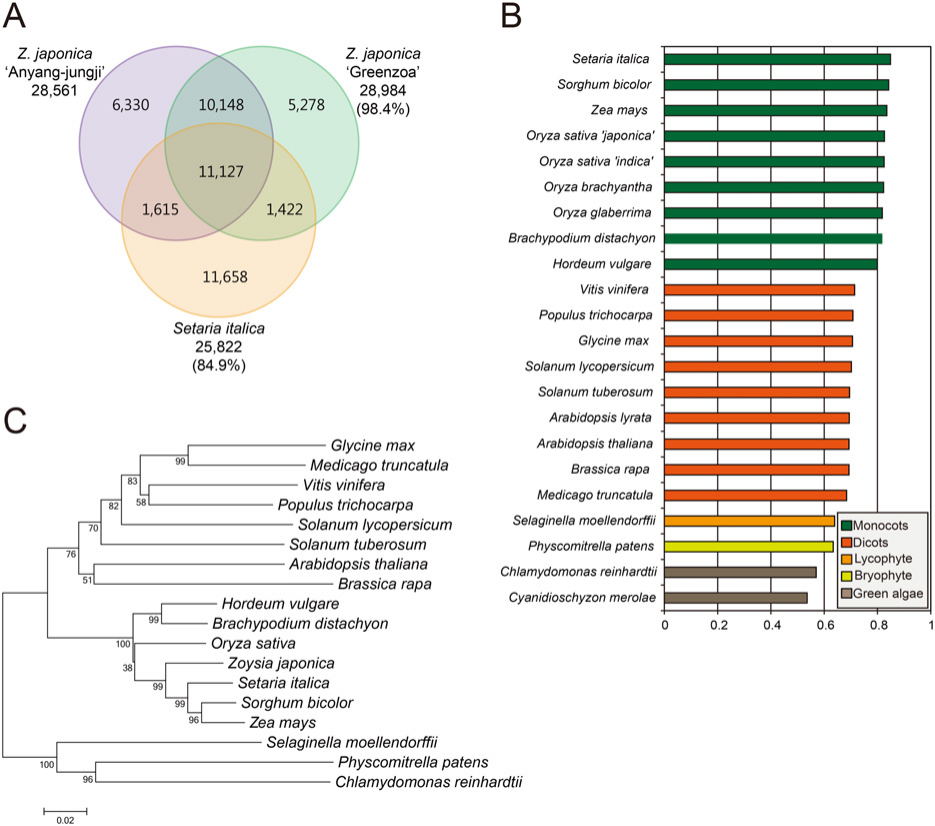
Phylogenetic relationship of *de novo* assembled zoysiagrass transcriptome to other plant species. (A) Transcript homology of two zoysiagrass cultivars and *Setaria italica*. Venn diagram shows the number of orthologous transcripts (E-value < 1.0e^-15^). The percentage in the brackets indicates the degree of transcriptome homology relative to AJ. (B) Relative similarity of zoysiagrass transcriptome with other plants. The zoysiagrass unigenes annotated with the NR database were matched by TBLASTX hit with E-value < 1.0E^-15^. (C) Phylogenetic tree based upon the *β-ACTIN* sequences from various plant species. The tree was constructed by using the maximum-likelihood method with MEGA 5.2 based on the ClustalX-generated multiple sequence alignment. The topological stability of the tree was evaluated by 1,000 bootstrap replications, and the bootstrapping percentage values are indicated by the numbers at the tree nodes. The GenBank accessions of *β-ACTIN* sequences used for phylogenetic analysis are listed in S4 Table.

From comparative transcriptome analysis between AJ and GZ, we obtained orthologous gene sets that were composed of 21,275 transcript pairs, where over 98.4% of GZ transcripts were identical to AJ transcripts at the nucleotide level (Fig 2A; S3 Table). As expected, the zoysiagrass transcriptome displayed relatively high similarities to those from several monocot species (Fig 2B). Among monocot species, the closest relative of *Z*. *japonica* appears to be *Setaria italica*, with approximately 84.8% of transcripts displaying significant homology to zoysiagrass transcriptome, and the next closest was *Sorghum bicolor* (Fig 2B). We also compared the nucleotide sequence of zoysiagrass *ACTIN* with its homologs from various plant species, and their phylogenetic relationship was established (Fig 2C; S4 Table). Consistently with transcriptome homology, zoysiagrass *ACTIN* is most similar to that of *S*. *italica*, *S*. *bicolor*, and other monocot plants. These data support that our zoysiagrass transcriptome was precisely assembled, and also demonstrate that zoysiagrass is genetically closest to *S*. *italica* among monocot species.

### Gene ontology (GO) term analysis and profiling of differentially expressed genes (DEGs)

Functional annotation of zoysiagrass transcripts was performed using Blast2GO [24]. From a complete set of *de novo* assembled zoysiagrass transcripts, 17,040 were assigned for their functions (S5 Table). They were classified into 44 major GO terms, where both AJ and GZ transcriptomes show very similar GO term distributions (S4A Fig).

**Fig 3:**
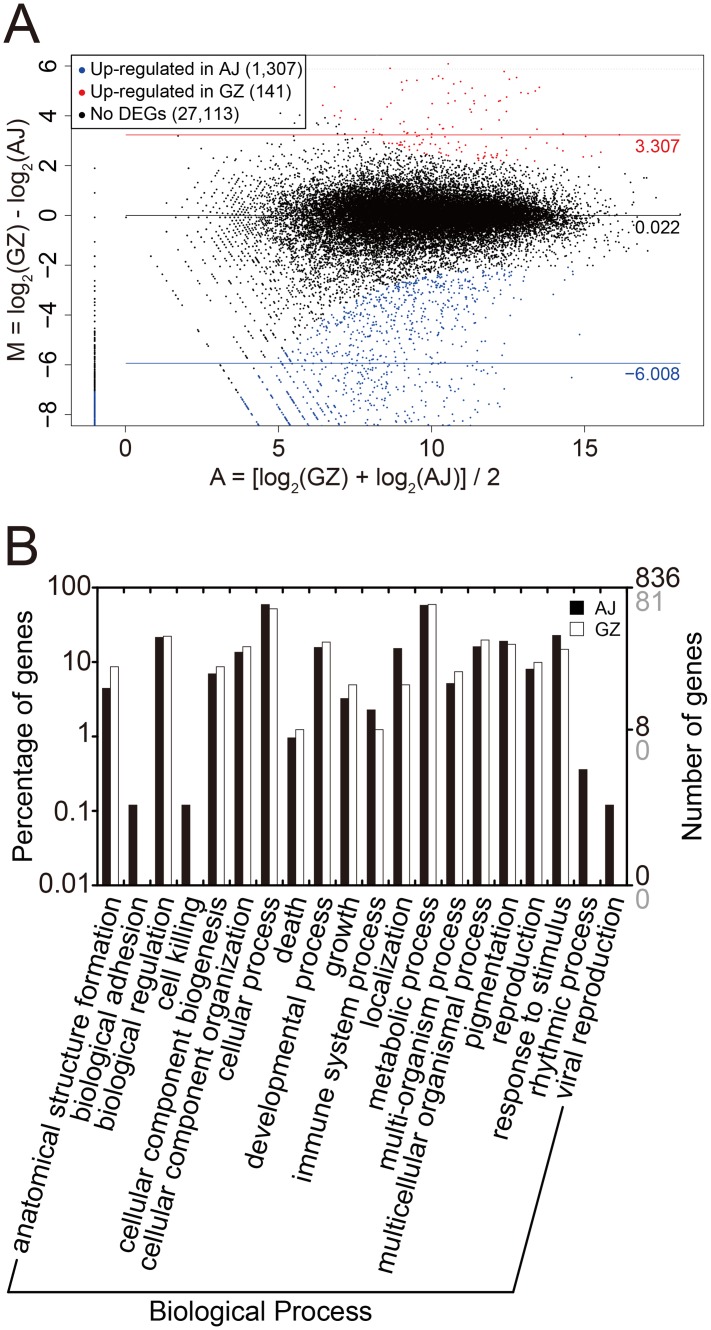
Differentially expressed genes between AJ and GZ spike tissues. (A) MA plot of differentially expressed transcripts identified in AJ and GZ. The X axis represents the mean expression level, and the Y axis represents the log_2_ fold-change of GZ transcripts over AJ. Red and blue dots represent differentially expressed genes (DEGs) that are significantly abundant in GZ and AJ, respectively, at p-value < 0.05. Horizontal lines and the values to the right represent the average M-values of corresponding groups of DEGs and non-DEGs. (B) Gene ontology (GO) classification of the DEGs. GO terms of *Z*. *japonica* unigenes are based on significant hits against the NR database. The right *y*-axis indicates the number of upregulated genes in AJ (black) and GZ (grey), respectively. The left *y*-axis indicates the percentage of each group from the total.

When expression levels of all orthologous transcript pairs were compared between AJ and GZ (Fig 2A; S3 Table), a total of 1,448 non-redundant transcript pairs were identified as significant DEGs using DESeq (p<0.01) (Fig 3A), where 1,307 AJ transcripts were more abundant than in GZ, whereas only 141 transcripts were less abundant. Among them, 836 upregulated DEGs and 81 downregulated DEGs were assigned to GO terms with 39 different categories (Pearson’s chi-squared test, p<0.05) (Fig 3B and S4 Fig). Notably, we found that a significant number of DEGs were assigned to GO term ‘pigmentation’ (GO:0043473), suggesting that these DEGs may be responsible for differential anthocyanin accumulation between AJ and GZ spikes (Fig 3B). To understand the genetic machinery of differential anthocyanin accumulation between AJ and GZ, we next focused on expression profiles of anthocyanin biosynthesis-related genes.

### Anthocyanin biosynthesis genes in zoysiagrass

**Fig 4:**
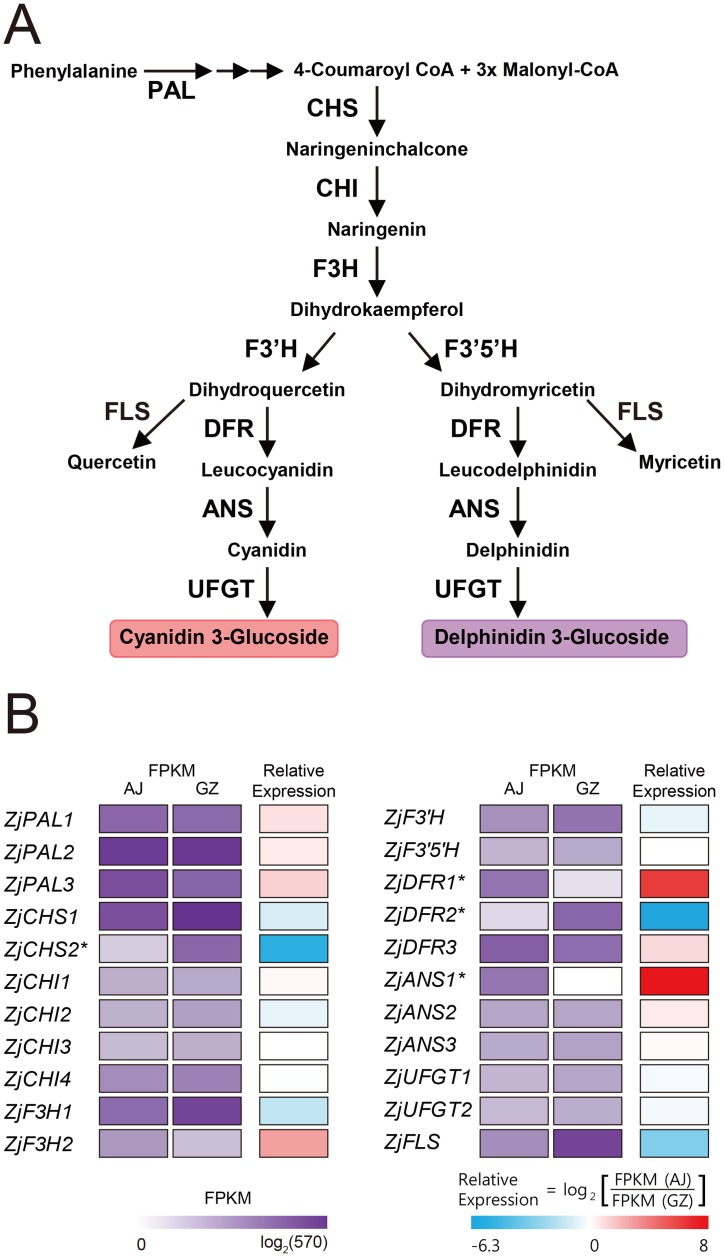
Zoysiagrass transcripts involved in general anthocyanin biosynthesis pathway. (A) A schematic view of the core anthocyanin biosynthesis pathway. ANS, anthocyanidin synthase; CHS, chalcone synthase; DFR, dihydroflavonol reductase; F3H, flavanone 3-hydroxylase; F3’H, flavonol 3’-hydroxylase; F3’5’H, flavonol 3’,5’-hydroxylase; FLS, flavonol synthase; PAL, phenylalanine ammonia lyase; UFGT, UDP-glucose:flavonoid 3-*O*-glucosyltransferase. (B) Heatmap of digital expression of candidate transcripts related to anthocyanin biosynthesis pathway in zoysiagrass. The log2-transformed FPKM values and relative expression (AJ vs. GZ) values are represented by the color map. Asterisks indicate statistical DEGs.

The anthocyanin biosynthesis pathway is branched from the general phenylpropanoid pathway that is carried out by sequential actions of a number of enzymes such as CHS, CHI, F3H, DFR, ANS, and several GTs [25]. Major genes involved in the anthocyanin biosynthesis pathway have been characterized in several plant species, and the overall pathway appears to be highly conserved in angiosperms. Therefore, we postulated that anthocyanin biosynthesis in zoysiagrass also takes place in a similar manner to other *Gramineae* species (Fig 4A).

Accumulation of anthocyanins such as cyanidin and petunidin in AJ spikes led us to investigate whether different coloration between AJ and GZ resulted from differential expression of anthocyanin biosynthesis genes. According to the Blast2GO annotation, 22 candidates were predicted to encode anthocyanin biosynthesis enzymes in zoysiagrass (Fig 4B; S6 Table). As showed in Fig 4B, expression levels of *PAL*s, *CHI*s, *F3H*s, and *UFGT*s were not significantly different between AJ and GZ. Interestingly, the expressions of *ZjDFR1* and *ZjANS1* were remarkably high in AJ relative to GZ, whereas the expressions of *ZjCHS2*, *ZjDFR2*, and *ZjFLS* were significantly low in AJ (S7 Table). These findings suggest that *ZjDFR1* and *ZjANS1* are strong candidates leading to anthocyanin biosynthesis and purple pigmentation in AJ, and that the lack of their expression, or little if any, may result in the failure of anthocyanin accumulation in GZ.

### Spatiotemporal expression of anthocyanin biosynthesis genes in zoysiagrass

**Fig 5:**
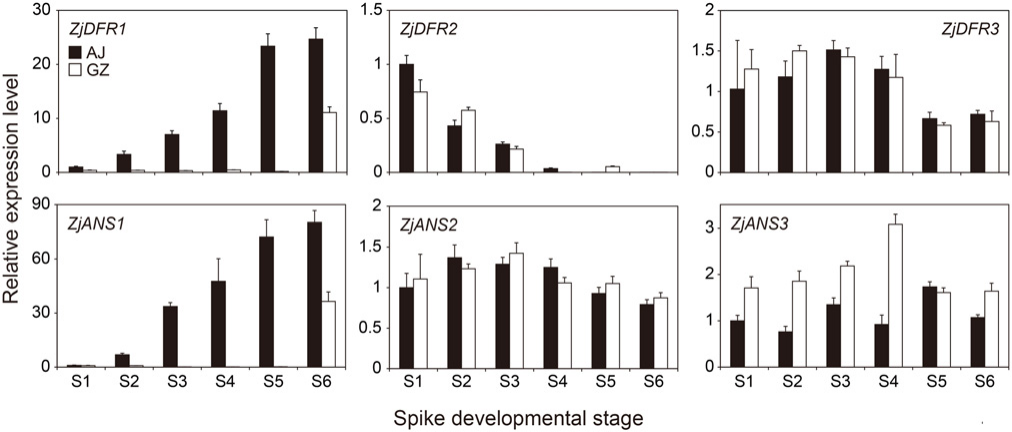
Expression levels of *DFR* and *ANS* genes in AJ and GZ spikes at different developmental stages. The relative expression level of each transcript was determined by quantitative RT-PCR, in which all values are normalized relative to the mean abundance of *β-ACTIN*. The expression levels are presented relative to the AJ gene abundance at stage S1. Bars represent means ± SD from triplicate biological repeats.

To verify the expression levels of DEGs identified from transcriptome analysis, we performed quantitative RT-PCR (qRT-PCR) on AJ and GZ spike tissues at six developmental stages (Fig 5 and S5 Fig). In AJ, in accordance with the increasing level of anthocyanin pigmentation (Fig 1A), expressions of both *ZjDFR1* and *ZjANS1* significantly increased as the spike developed, whereas their expression was contrastingly low in developing GZ spikes until stage S5 (Fig 5). This also supports our transcriptome data, in which both *ZjDFR1* and *ZjANS1* transcripts were found to be highly abundant only in purple AJ spikes (Fig 4B). By contrast, *ZjANS2*, *ZjANS3*, *ZjDFR2*, and *ZjDFR3* did not display meaningful expression patterns throughout the developmental stages (Fig 5). For example, *ZjANS2* and *ZjDFR3* were constantly expressed at all stages, but *ZjDFR2* expression was highest in early spikelets and gradually decreased in both AJ and GZ spikes toward maturation (Fig 5). Therefore, we hypothesize that both *ZjDFR1* and *ZjANS1* are developmentally regulated to control the synthesis of anthocyanin pigments in purple zoysiagrass spikes but the absence of their expression leads to no pigmentation in the green spike.

Tissue-specific expressions of zoysiagrass anthocyanin synthesis genes were examined in several other tissues such as stolon, young and old leaves, and root. As in the spike, *ZjDFR1* was also highly expressed in the AJ stolon, another tissue showing strong purple pigmentation, while low levels of expression were observed in the other tissues (S6 Fig). *ZjANS1* was also expressed in the AJ stolon, although not as much as in the AJ spike, but its expression was rarely detectable in other tissues of AJ and GZ (S4 Fig). Three *PAL* genes (*ZjPAL1~3*) were highly expressed in the AJ root, but their expression probably had no relationship with tissue-specific anthocyanin biosynthesis, as the AJ root did not accumulate purple pigments. Our quantitative expression analysis revealed that besides *ZjDFR1* and *ZjANS1*, no other anthocyanin biosynthesis-related genes were likely involved in the tissue-specific pigmentation in zoysiagrass (S6 Fig).

### *ZjDFR1* and *ZjANS2* confer different anthocyanin accumulation in zoysiagrass

Several anthocyanin biosynthesis genes in angiosperm are reported to exist in multiple copies. In particular, *DFR* and *ANS* homologs are categorized into several phylogenetic subclades and each subclade is expected to have different functional activity in the pathway [26, 27]. In an effort to assess the distinct functions of zoysiagrass DFR and ANS in anthocyanin biosynthesis, we analyzed the phylogenetic relationships of these proteins with other homologs that have been previously characterized (S8 Table). When compared with DFR protein sequences from 11 other plant species, ZjDFR1 is assigned to the same group with other DFR homologs, whereas ZjDFR2 and ZjDFR3 were distantly related (S7A Fig; S8 Table). Similarly, ZjANS1 is grouped into the same clade with 10 other ANS homologs, but ZjANS2 and ZjANS3 are in the outgroup (S7B Fig; S8 Table). These results indicate that ZjDFR1 and ZjANS1 have similar structures and functions, and thus, their proper spatiotemporal expression may be required for the anthocyanin biosynthesis in zoysiagrass.

**Fig 6:**
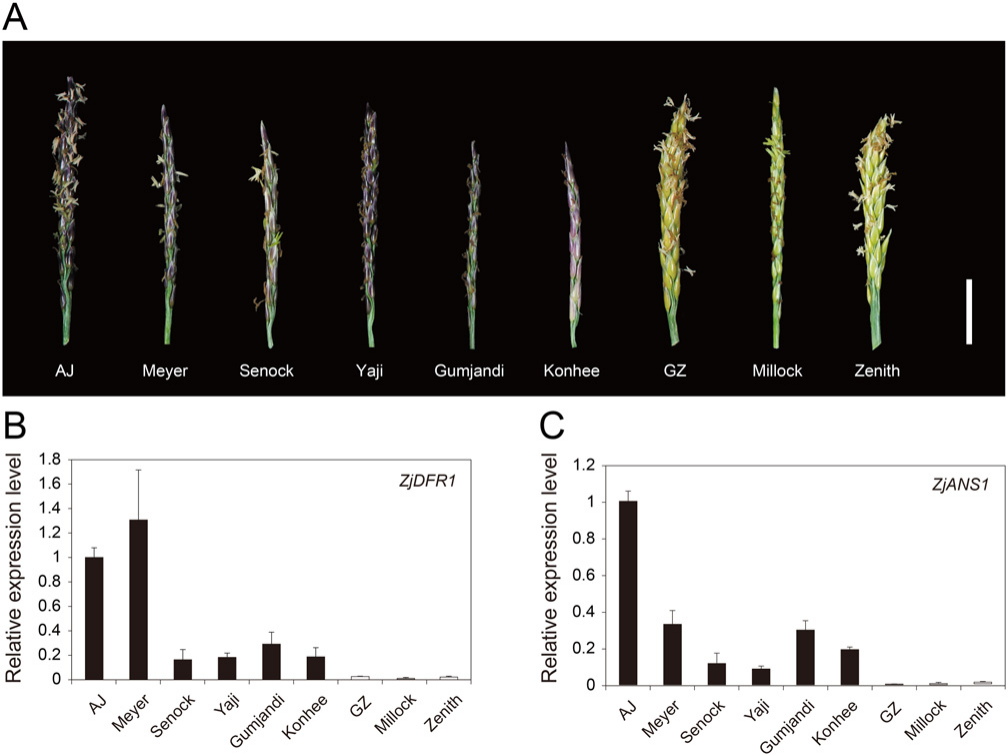
Analysis of *ZjDFR1* and *ZjANS1* expression among zoysiagrass cultivars. (A) Representative spikes of nine zoysiagrass cultivars at developmental stage S6. The scale bar represents 10 mm. Expression levels of *ZjDFR1* (B) and *ZjANS1*(C) genes in spike tissues determined by quantitative RT-PCR. All values are normalized relative to the mean abundance of *β-ACTIN*. Bars represent means ± SD from triplicate biological repeats.

Notably, differential anthocyanin accumulation in the spike and stolon tissues is also observed in other zoysiagrass cultivars. Therefore, we investigated whether tissue-specific upregulation of *ZjDFR1* and *ZjANS1* was a common phenomenon in zoysiagrass plants with purple-colored spikes and stolons. Nine zoysiagrass cultivars with different spike and stolon colors were collected and analyzed for the anthocyanin content (Fig 6A and S8 Fig; S9 Table). HPLC analysis revealed that significant amounts of anthocyanins accumulated in the spikes of six purple-pigmented cultivars, whereas no anthocyanin was detected in the other three green colored ones (S9 Fig). In addition, expression studies demonstrated that although expression levels of *ZjDFR1* and *ZjANS1* varied among purple-pigmented cultivars, the green stolon cultivars had significantly low levels of both transcripts (Fig 6B, 6C, and S8 Fig). These observations strongly suggest that differential regulation of *ZjDFR1* and *ZjANS1* at the transcription level determines the rate of anthocyanin accumulation in zoysiagrass, which is expressed as distinct pigmentation in the spike and stolon tissues.

### Isolation of genes encoding DFR and ANS protein from zoysiagrass

To verify whether differential anthocyanin pigmentation is exclusively determined at the transcription level, we compared the structures of *ZjDFR1* and *AjANS1* genes between AJ and GZ. We revealed that the *ZjDFR1* gene consists of 1,458 bp of open reading frame (ORF) with 1,113 bp of CDS, whereas *ZjANS1* contains only 1,179 bp of ORF with no intronic sequences (S10 Fig). Overall structures of these genes are highly similar to those of other monocot species such as maize, rice, and sorghum (S10 Fig). Furthermore, they share more than 98% of sequence identity at the amino acid level among different zoysiagrass cultivars (S11 Fig). Interestingly, each of *ZjDFR1* and *ZjANS1* was found to have exactly the same coding sequence between AJ and GZ (S12 Fig), indicating that structures and functions of their gene products are the same, and therefore, the regulatory elements for transcriptional control might be more important for differential anthocyanin biosynthesis between AJ and GZ.

In order to find out the differences in the regulatory region, if any, we obtained and compared the sequences of 427 and 475 bp upstream of translation start sites of *ZjDFR1* and *ZjANS1*, respectively (S13 Fig). As the same as in the ORF regions, each of these genes was found to have the identical sequence in the promoter region between AJ and GZ (S12 Fig). Notably, they were predicted to have several *cis*-regulatory sequences such as an ABA response element (ABRE), G-box, and MYB binding site (MBS) motifs (S14 Fig), suggesting that differential regulation of these genes between AJ and GZ spikes may be under a control of certain transcription factors.

However, we could not rule out the possibility of epigenetic modifications for transcriptional control, and thus, we looked for differentially methylated regions (DMRs) at the promoters of AJ and GZ spike tissues by bisulfite sequencing (BS-seq). As shown in S15 Fig, the promoter regions of both *ZjDFR1* and *ZjANS1* have nearly identical DNA methylation patterns between AJ and GZ. For example, all cytosine residues in three different sequence contexts (CG, CHG, and CHH, where H = A, C, or T) were hypomethylated in both AJ and GZ *ZjDFR1* genes. By contrast, the *ZjANS1* gene was found to have common CG and CHG methylation sites in both AJ and GZ spike tissues. However, the lack of DMRs between AJ and GZ strongly suggests that differential tissue-specific expression of *ZjDFR1* and *ZjANS1* is primarily regulated by genetic control rather than by epigenetic factors.

### Dihydroflavonol reductase and leucocyanidin oxygenase activities of recombinant ZjDFR1 and ZjANS1 proteins

Although our transcriptome and expression studies strongly suggest that ZjDFR1 and ZjANS1 primarily regulate tissue-specific anthocyanin pigmentation in purple zoysiagrass cultivars, it cannot be ruled out that they may have other catalytic functions, or some other genes not revealed by our analysis may encode proteins that actually catalyze essential steps in the anthocyanin biosynthesis pathway. Therefore, we expressed recombinant ZjDFR1 and ZjANS1 proteins in *E*. *coli* (S16 Fig) and performed *in vitro* studies to reveal their biochemical activities. In the conventional anthocyanin biosynthesis pathway, DFR catalyzes the conversion of dihydroflavonols to leucoanthocyanidins, and in the next step, ANS acts to convert leucoanthocyanidins into anthocyanidins (Fig 4A; [28]). Bacterial cell extracts expressing recombinant ZjDFR1 protein were incubated with dihydroflavonols such as dihydromyricetin (DHM), dihydrokaempferol (DHK), and dihydroquercetin (DHQ) and the reaction products were analyzed by HPLC (S17 Fig). As compared to the HPLC profile of control reaction, an additional peak was observed when each dihydroflavonol was reacted with bacterial cell extracts expressing ZjDFR1 (S17 Fig). These peaks are supposedly leucoanthocyanidins such as leucodelphinidin, leucocyanidin, and leucopelargonidin produced by ZjDFR1, as the subsequent LC-MS analysis revealed the appearance of another close peak with a two mass unit difference from substrate (dihydroflavonol) due to the addition of two hydrogen atoms by DFR activity (S18 Fig). No reaction product was observed when naringenin was used as substrate, indicating that ZjDFR1 activity is specific to dihydroflavonols (S18 Fig). Notably, when dihydroflavonols were reacted with the mixture of bacterial cell extracts expressing ZjDFR1 and ZjANS1, respectively, another peaks were conspicuously detected (S17 Fig). These peaks were found to correspond to delphinidin, cyanidin, and pelargonidin, suggesting that ZjANS1 is able to catalyze the conversion of leucoanthocyanidins into anthocyanidins.

## Discussion

Anthocyanins are one of the major color pigments in plants, which have diverse physiological functions [15]. However, from an aesthetic point of view, purple coloration in spikes and stolons by anthocyanin accumulation brings about blotchy appearance mixed with otherwise uniform green colors, which often lowers the commercial values of zoysiagrass as a lawn. Therefore, developing the varieties with even green color is one of the breeding goals in zoysiagrass [4, 29].

In this study, we compared the anthocyanin profiles between two zoysiagrass cultivars AJ and GZ, whose spikes and stolons develop purple and green colors, respectively (Fig 1A and S1 Fig). Detection of cyanidin and petunidin as major aglycone forms of anthocyanins in developing spikes of AJ, but not in green spikes of GZ (Fig 1B), indicates that differential anthocyanin biosynthesis occurs between the two cultivars in a tissue-specific manner.

*De novo* transcriptome analysis on developing spike tissues identified > 28,000 unigenes expressed in AJ and GZ (Fig 2), where approximately 5% of genes were found to be differentially expressed (Fig 3A). Interestingly, two anthocyanin biosynthesis genes *ZjDFR1* and *ZjANS1* were highly upregulated in purple AJ spike tissues (Figs 4B and 5). Further expression analysis on purple and green spike tissues from a variety of zoysiagrass cultivars revealed a strong correlation between purple pigmentation and expression levels of *ZjDFR1* and *ZjANS1* (Fig 6). Our *in vitro* study confirms that ZjDFR1 and ZjANS1 have essential biochemical activities to convert dihydroflavonols into leucoanthocyanidins and leucoanthocyanidins into anthocyanidins, respectively (S17 Fig). This strongly implies that anthocyanin biosynthesis and corresponding purple pigmentation in spike and stolon tissues in zoysiagrass are primarily regulated by the upregulation of *ZjDFR1* and *ZjANS1* genes.

Deep purple coloration of developing AJ spikes and stolons are due to the accumulation of cyanidin (Fig 1), presumably in the form of cyanidin-3-O-glucoside. Its accumulation is significantly larger than that of petunidin (Fig 1B). This suggests that dihydroquercetin, a precursor of leucocyanidin, is more preferentially formed from dihydrokaempferol (Fig 4). Supporting this idea, our transcriptome analysis revealed that the expression of F3’H that catalyzes the conversion of dihydrokaempferol to dihydroquercetin is significantly higher than F3’5’H in developing spikes (Fig 4B), suggesting that the downstream synthesis of leucocyanidin and cyanidin primarily takes places in purple-pigmented zoysiagrass tissues. By contrast, the synthesis of petunidin, which requires the supply of dihydromyricetin that is produced by F3’5’H, is relatively small. Considering dihydrokaempferol serves as a common precursor for dihydroquercetin and dihydromyricetin, whose syntheses are catalyzed by F3’H and F3’5’H, respectively, upregulation of F3’H may divert the metabolic flow at the branch point to the synthesis of cyanidin pigments rather than petunidin.

The importance of ANS and DFR in anthocyanin biosynthesis has been reported in both monocots and dicots. In maize, for example, the *a1* and *a2* genes encode DFR and ANS, respectively, and they are essential for anthocyanin pigmentation in the aleurone layer of the kernel [30–33]. In Arabidopsis, *TRANSPARENT TESTA3* (*TT3*) and *TT18* encode corresponding enzymes, respectively, and their mutants have a pale yellow seed coat due to a lack of condensed tannins (proanthocyanidins) [34, 35].

In line with the previous studies, we found that putative *ANS* and *DFR* genes in zoysiagrass were also upregulated according the level of anthocyanin biosynthesis. For example, both *ZjDFR1* and *ZjANS1* genes are highly expressed in developing AJ spikes, whereas their expressions are relatively low in green GZ tissues (Fig 5). However, there appears to be no correlation between expression of other *ANS* and *DFR* genes (*ZjDFR2*, *ZjDFR3*, *ZjANS2*, and *ZjANS3*) and anthocyanin biosynthesis in developing purple AJ spikes (Fig 5). This indicates that a specific set of anthocyanin biosynthesis genes such as *ZjDFR1* and *ZjANS1* are developmentally regulated in a tissue-specific manner. Besides purple pigmentation in spikes and stolons, mature seeds of AJ have a dark brown seed coat, whereas those of GZ have pale brown colors (S19 Fig). This is due probably to differential accumulation of condensed tannins between AJ and GZ seed coats, which is reminiscent of the seed coat color differences between wild type and *tt* mutants in *Arabidopsis* [34, 35]. Therefore, it is intriguing in that both monocots and dicots are evolutionarily divergent but probably have maintained similar mechanisms to regulate flavonoids biosynthesis and their accumulation in the seeds.

How are these *ZjDFR1* and *ZjANS1* genes specifically upregulated only in purple AJ spike and stolon tissues? We revealed that the coding sequences of *ZjDFR1* and *ZjANS1* genes from AJ and GZ are identical to each other (S12 Fig). This implies that functions of these gene products are not determined by their structures but rather by a transcriptional control. The regulatory sequences upstream of the translational start sites also do not display significant differences, and our bisulfite sequencing analysis demonstrated that promoter regions of *ZjDFR1* and *ZjANS1* have similar levels and patterns of DNA methylation between AJ and GZ (S15 Fig). This indicates that differential expression of *ZjDFR1* and *ZjANS1* between AJ and GZ is not determined by DNA methylation, and thus the epigenetic control, at least DNA methylation, is unlikely a central mechanism for their transcriptional regulation.

Plants generally respond to developmental and environmental signals by regulating specific transcription factors. In recent years, MYB transcription factors have been extensively studied for their roles in the regulation of pigmentation in plants [36, 37]. It is known that R2R3-type MYB proteins and the MYB-bHLH-WD40 complex activate the transcription of early (*CHS*, *CHI*, *F3’H*, and *FLS*) and late (*DFR*, *ANS*, and *ANR*) flavonoid biosynthesis genes, respectively [38]. The light-dependent anthocyanin accumulation in the skin of apple fruit can be also explained by the activation of a MYB transcription factor. For instance, MdMYB1, which controls apple anthocyanin pathway genes, is repressed under darkness by interacting with MdCOP1 and subsequent ubiquitin-dependent protein degradation [28]. Interestingly, two zoysiagrass *MYB* gene (*ZjMYB1* and *ZjMYB2*) transcripts were found to be more abundant in purple AJ spike and stolon tissues than in green GZ tissues (S6 Fig), and both *ZjDFR1* and *ZjANS1* contain several *cis*-regulatory elements such as ABRE, G-box, MBS, and AP2 binding motifs which might serve as putative MYB binding sites (S14 Fig). These findings suggest that two MYB transcription factors ZjMYB1 and ZjMYB2 may specifically activate late flavonoid biosynthesis genes such as *ZjDFR1* and *ZjANS1*, albeit their direct role in anthocyanin biosynthesis regulation awaits further investigation.

In summary, we demonstrate the efficiency of *de novo* transcriptome analysis in plant species whose genome information is scarcely available. We successfully identified a number of anthocyanin biosynthesis genes and correlated their expression patterns with tissue-specific pigmentation in various zoysiagrass cultivars. This study will not only provide a mechanistic insight into the regulation of flavonoid biosynthesis in *Zoysia* plants, but also facilitate extensive genome studies in related species.

## Materials and Methods

### Plant materials

Zoysiagrass (*Zoysia japonica*) cultivars ‘Anyang-Jungji’ (AJ) and ‘Greenzoa’ (GZ) were obtained from Fungi and Plant (FnP) Cooperation (Jeungpyeong-gun, Chungcheongbuk-do, Korea). The two cultivars were grown in the greenhouse with 16 h light/8 h dark cycles, 26/22°C (day/night) temperatures, and 60% relative humidity. Other seven zoysiagrass cultivars Meyer, Senock, Yaji, Gumjandi, Konhee, Millock, and Zenith were grown in the field of Experimental Farm of Seoul National University (Suwon, Gyeonggi-do, Korea).

### RNA extraction and RNA-seq

Total RNAs were extracted from spike, stolon, young/old leaf and root tissues in the TRIzol reagent (Life Technologies) according to the manufacturer’s protocol. For transcriptome analysis, total RNA from AJ and GZ spike tissues was subjected to RNA-seq. Sequencing was performed on an Illumina HiSeq 2000 system in the National Instrument Center for Environmental Management (NICEM, http://nicem.snu.ac.kr). The raw sequences were deposited in the NCBI/EBI/DDBJ Short Read Archive (Accession number: DRA001679).

### *De novo* transcriptome assembly

The raw reads were filtered by removing the adapter sequences, empty reads, low quality reads, and the reads with more than 20% Q < 20 bases. Transcriptome *de novo* assembly was carried out with three assemblers such as CLC Genomics Workbench (ver. 3.7.1), Velvet (ver. 1.1.04)-Oases (ver. 0.1.21), and Trinity (release 20110519) [39, 40] with various k-mer lengths. A default k-mer value (25-mer) was used for assembly with CLC. For Velvet-Oases and Trinity, we applied different k-mer values to get the best results (from 21 to 79 for Velvet-Oases and 25 to 33 for Trinity). As Oases does not cluster assembled contigs, we used CD-HIT-EST to cluster the contigs with identity more than 90% and coverage of 100% [41]. All resulting data sets were merged into a single assembly by collapsing identical or near-identical contigs into single unigenes. Assembled transcriptomes are available at http://epigenome.snu.ac.kr/.

### Gene ontology (GO) annotation

The unigenes were identified by sequence similarity comparison against NCBI non-redundant (NR) protein database (http://www.ncbi.nlm.nih.gov) by running BLAST with a cut-off E-value of 10^-6^. Blast2GO was used to obtain GO annotation of unigenes based on BLASTX hit against NR database with a cut-off E-value of 1.0e^-6^ (ver. 2.6.5; [42]). Obtained GO terms were classified and plotted using WEGO software [43].

### Phylogenetic study

A total of 22 plant transcriptome data sets (9 monocots, 9 dicots, 2 bryophytes, and 2 green algae) were obtained from the Ensembl Plants database (http://plants.ensembl.org/). *De novo* assembled AJ transcripts were aligned against these transcriptome sequences using TBLASTX program with a cut-off E-value of 1.0e^-15^. To assess the phylogenetic relationship among zoysiagrass and other plant species, we performed phylogenetic analysis using *ACTIN* sequences derived from 22 different plant species (S4 Table). Orthologous transcripts of *ZjDFR1* and *ZjANS1* were obtained by TBLASTX search with a cut-off E-value of 1.0e^-15^, and sequences were aligned and compared with ClustalΧ (ver. 2.1; [44]). The phylogenetic tree was constructed by MEGA5.2 [45] using Poisson model and with 1,000 bootstrap replications for each branch. Sequence information of transcripts used in phylogenetic analysis is provided as S1 File.

### Quantitative real-time PCR (qRT-PCR) analysis

A total of 1–2 μg RNA was treated with RNase-Free DNase Set (QIAGEN) to remove contaminating DNA and then subjected to cDNA synthesis using the SuperScript II RT Kit (Life Technologies) according to the manufacturer’s instructions. qRT-PCR was performed on a Rotor-Gene Q real-time PCR system (QIAGEN). QuantiFast SYBR Green PCR master mix (QIAGEN) was used for amplification. Zoysiagrass *β-ACTIN* (GU290545.1) sequence was used as an internal control to measure the relative amount of transcripts. Information on oligonucleotide sequences for qRT-PCR analysis is listed in S10 Table.

### 5’-/3’-RACE and thermal asymmetric interlaced (TAIL)-PCR

To obtain full-length sequences of *ZjDFR1* and *ZjANS1*, the region outside the contig was extended with SMARTer RACE cDNA Amplification Kit (Clontech Laboratories) according to the manufacturer’s protocol. To obtain the sequence of regulatory region upstream of transcription start site, TAIL-PCR [46] was performed. The *cis*-acting regulatory element at the promoter region was predicted by homology search against the PlantCARE database [47]. Information on oligonucleotide sequences for RACE and TAIL-PCR is provided in S10 Table.

### Local bisulfite sequencing (BS-seq)

Genomic DNA was extracted from AJ and GZ spikes at stages S5 and S6 [48]. The sodium bisulfite treatment and following DNA purification were carried out using EpiTect Bisulfite Kit (QIAGEN) according to the manufacturer’s protocol. Information on oligonucleotide sequences for BS-seq analysis is provided in S10 Table.

### Expression of ZjDFR1 and ZjANS1 in *E*. *coli*

Cloning and expression of ZjDFR1 and ZjANS1 genes in *E*. *coli* were performed according to the previous study with minor modifications [49]. Briefly, the cDNA fragments of ZjDFR1 and ZjANS1 were cloned into the pLM302 vector and the constructs were transformed into *E*. *coli* Rosetta2 (DE3) strain (EMD Millipore). Protein expression was induced with 0.1 mM IPTG at 16°C overnight with shaking. Cells were harvested by centrifugation and the pellet was resuspended in 10 mL of lysis buffer (50 mM Tris-HCl, pH 7.4, 100 mM NaCl, 10% glycerol, 0.1 mM dithiothreitol, 0.1 mM PMSF). After sonication and centrifugation, the supernatant was collected and used for *in vitro* analysis.

### *In vitro* assay of ZjDFR1 and ZjANS1

*In vitro* biochemical assay of ZjDFR1 and ZjANS1 was performed according to the previous study with minor modifications [50]. As substrate, naringenin and dihydroflavonols DHQ, DHK, and DHM (Sigma-Aldrich) were dissolved in methanol at 1 mg/mL concentration. For ZjDFR1 reaction, each substrate (10 μg) was reacted with *E*. *coli* cell extracts (100 μg of total protein) expressing ZjDFR1 in the reaction buffer (100 mM Tris-HCl, pH 7.0, 1 mM NADPH) at 30°C for 30 min. Reactions were terminated by adding 1 mL of ethyl acetate, and the extracts were evaporated with a stream of nitrogen gas. Remaining solid compounds were dissolved in methanol and subjected to HPLC analysis. The ZjANS1 assay followed the same procedure by using equal amounts of *E*. *coli* cell extracts expressing ZjDFR1 and ZjANS1, respectively, in the same reaction.

### HPLC analysis

To detect major anthocyanidins derived from anthocyanins, HPLC analysis was performed according to the previous report with minor modifications [22]. Briefly, anthocyanin pigments were extracted with a solvent mixture of acetone:methanol:water:formic acid (40:40:20:0.1, v/v/v/v). Extracts were filtered through Sep-Pak C18 cartridge (Waters Scientific, Ontario, CA). For hydrolysis of anthocyanins to aglycones, 3 mL of 2N HCl in 50% (v/v) aqueous methanol was added to the sample powder. After incubation at 100°C for 1 h, the extracts were injected onto the Eclipse ZOBRAX XDB-C18 Rapid Resolution Threaded Column (4.6 × 150 mm, 5 μm; Agilent Technologies) on an UltiMate 3000 HPLC system (Thermo Scientific), using delphinidin chloride, cyanidin chloride, peonidin chloride, malvidin chloride (Sigma-Aldrich), and petunidin chloride (EXTRASYNTHESE) as standards. Quantification of anthocyanin aglycone was determined at the wavelength of 520 nm. For detection of leucoanthocyanidins and anthocyanidins from in vitro assay, the samples were separated in the Inno C18 column (4.6 mm × 250 mm, 5 μm; Innopia, Korea) on an UltiMate 3000 HPLC system (Thermo scientific) with detection at 280 nm.

### LC-MS analysis

LC-MS analysis was performed using a ThermoFinnigan LCQ Deca XP plus ion trap mass spectrometer (Thermo Scientific, USA) with an ESI interface at positive ion mode scanned from *m/z* 100 to 600. The capillary temperature was maintained at 275°C, the source voltage was 5 kV, and the capillary voltage was set at 45 V.

## Supporting Information

S1 Fig Field-grown phenotypes of two zoysiagrass cultivars used in this study. Anyang-jungji (A) and Greenzoa (B). Arrows indicate creeping stolons.
(TIF)

S2 Fig HPLC analysis of anthocyanin aglycones present in the AJ and GZ spikes at six developmental stages. The chromatograms were recorded at 520 nm. a, delphinidin; b, cyanidin; c, peonidin; d, petunidin; e, malvidin.
(TIF)

S3 Fig HPLC analysis of anthocyanins in the AJ spikes. The chromatograms were recorded at 475 nm. 1, delphinidin-3-O-glucoside; 2, cyanidin-3-O-glucoside; 3, pelargonidin-3-O-glucoside; 4, peonidin-3-O-glucoside; 5, malvidin-3-O-glucoside; 6, luteolinidin.
(TIF)

S4 Fig Gene ontology classification of zoysiagrass transcriptome. Gene ontology (GO) terms for each zoysiagrass transcripts were assigned based on significant TBLASTX hits (e-value < 1e-15) against the NR database. (A) The results are summarized in three main categories (biological process, molecular function, and cellular component) and 44 subcategories. (B) Gene ontology classification of DEGs between AJ and GZ.
(TIF)

S5 Fig Expression levels of anthocyanin biosynthesis genes at different developmental stages of AJ and GZ spikes. The relative expression level of each transcript was determined by qRT-PCR. All values are normalized relative to the mean abundance of *β-ACTIN* at each stage. Bars represent means ± SD from triplicate biological repeats.
(TIF)

S6 Fig Expression levels of anthocyanin biosynthesis genes in diverse tissues of AJ and GZ. The relative expression level of each transcript was determined by qRT-PCR. All values are normalized relative to the mean abundance of *β-ACTIN*. Bars represent means ± SD from triplicate biological repeats.
(TIF)

S7 Fig Phylogenetic trees of the DFR and ANS protein families. (A) Phylogeny of the DFR protein family. (B) Phylogeny of the ANS protein family. Am, *Antirrhinum majus*, At, *Arabidopsis thaliana*; Gh, *Gerbera hybrid*; Gt, *Gentiana triflora*; Hv, *Hordeum vulgare*; Lj, *Lotus japonicus*; Md, *Malus domestica*; Nt, *Nicotiana tabacum*; Os, *Oryza saiva*; Pc, *Pyrus communis*; Ph, *Petunia hybrid*; Rh, *Rosa hybrid*; Ta, *Triticum aestivum*; Vm, *Vaccinium macrocarpon*; Vv, *Vitis vinifera*; Zm, *Zea mays*. Accession numbers of the proteins analyzed are listed in the S8 Table.
(TIF)

S8 Fig Expression analysis of *ZjDFR1* and *ZjANS1* genes in stolon tissues of nine zoysiagrass cultivars. (A) Representative pictures of the stolon of nine zoysiagrass cultivars. The guide bar indicates 10 mm. (B and C) Expression patterns of *ZjDFR1* (B) and *ZjANS1* (C) at stolon by qRT-PCR. All values are normalized relative to the mean abundance of *β-ACTIN*. Bars represent means ± SD from triplicate biological repeats.
(TIF)

S9 Fig HPLC profiles of anthocyanins present in the stolon tissues of nine zoysiagrass cultivars. The chromatograms were recorded at 520 nm. Red and blue arrowheads indicate cyanidin-3-O-glucoside and malvidin-3-O-glucoside, respectively.
(TIF)

S10 Fig Schematic representations of exon-intron structures of DFR and ANS proteins derived from different species. Closed bars and lines represent exons and introns, respectively. Zj, *Zoysia japonica*; Si, *Setaria italica*; Sb, *Sorghum bicolor*; Zm, *Zea mays*; Bd, *Brachypodium distachyon*; At, *Arabidopsis thaliana*.
(TIF)

S11 Fig Sequence alignments of predicted ZjDFR1 (A) and ZjANS1 (B) proteins among nine zoysiagrass cultivars. (A) Arrowheads on ZjDFR1 indicate conserved amino acid residues in the hydroxysteroid dehydrogenase/DFR superfamily [51]. (B) Arrowheads on ZjANS1 indicate conserved His and Asp residues required for ferrous-iron coordination, and Arg for putative 2-oxoglutarate binding site [52].
(TIF)

S12 Fig Sequence alignments of *ZjDFR1* (A) and *ZjANS1* (B) CDS between AJ and GZ. (TIF)

S13 Fig Sequence alignments of the promoter regions of *ZjDFR1* (A) and *ZjANS1* (B) among nine zoysiagrass cultivars. (TIF)

S14 Fig Predicted *cis*-acting elements on the *ZjDFR1* and *ZjANS1* promoter regions. The bold ‘ATG’ indicates the translational start site. Predicted *cis*-regulatory elements are underlined and identified according to the PlantCare database [49]. ABRE, abscisic acid response element; MBS, MYB binding site; AP2, Apetala2.
(TIF)

S15 Fig DNA methylation profiles of *ZjDFR1* (A) and *ZjANS1* (B) promoter region from AJ and GZ. Numbers are from the translational start site. 5-methylcytosines in the CG (circle), CHG (triangle), and CHH (square) contexts were displayed by CyMATE [53]. Open and closed shapes indicate unmethylated and methylated cytosines, respectively. Primers used for bisulfite sequencing are listed in S12 Table.
(TIF)

S16 Fig Expression of recombinant ZjDFR1 and ZjANS1 proteins fused with an N-terminal maltose binding protein (MBP) fragment in *E*. *coli*. Total proteins were separated on a 10% SDS-PAGE gel and visualized by Coomassie Brilliant Blue staining. Expected molecular weights of MBP, MBP-ZjDFR1, and MBP-ZjANS1 are indicated to the right side of the panel. M, size marker.
(TIF)

S17 Fig HPLC analysis of leucoanthocyanidins and anthocyanidins produced by recombinant ZjDFR1 and ZjANS1 proteins in *E*. *coli*. The chromatograms were recorded at 280 nm. a, leucodelphinidin; b, leucocyanidin; c, leucopelargonidin.
(TIF)

S18 Fig LC-MS analysis of the reaction mixtures of dihydroflavonols incubated with *E*. *coli* cell extracts expressing ZjDFR1. Mass spectra obtained for bacterial cell extracts incubated with four substrates and the [M+] (*m/z*) values of the corresponding peaks: dihydromyricetin (DHM; *m/z* = 321), dihydroquercetin (DHQ; *m/z* = 305), dihydrokaempferol (DHK; *m/z* = 289), and naringenin (NRG; *m/z* = 273).
(TIF)

S19 Fig Seed colors of AJ (left) and GZ (right). The guide bar indicates 0.5 mm.
(TIF)

S1 File Alignment of ZjDFR1 and ZjANS1 homologs. (DOC)

S1 Table Summary of filtered and assembled RNA-seq data from AJ and GZ spike tissues. (DOCX)

S2 Table Summary of *de novo* assembled transcriptomes of AJ and GZ spike tissues using multiple assemblers. (DOCX)

S3 Table Orthologous gene sets between AJ and GZ. (XLSX)

S4 Table GenBank accession numbers of *ACTIN* sequences used for phylogenetic analysis. (DOCX)

S5 Table List of GO terms for each zoysiagrass contig. (XLSX)

S6 Table Zoysiagrass unigenes used for qRT-PCR analysis. (DOCX)

S7 Table FPKM values of anthocyanin biosynthesis genes in AJ and GZ. (DOCX)

S8 Table GenBank accession numbers of DFR and ANS homologous proteins derived from other plant species. (DOCX)

S9 Table Characteristics of several zoysiagrass cultivars used for HPLC and qRT-PCR analysis. (DOCX)

S10 Table Primer sequences used in this study. (DOCX)
