# Correction: *De Novo* Transcriptome Analysis to Identify Anthocyanin Biosynthesis Genes Responsible for Tissue-Specific Pigmentation in Zoysiagrass (*Zoysia japonica* Steud.)

**DOI:** 10.1371/journal.pone.0137943

**Published:** 2015-09-04

**Authors:** Jong Hwa Ahn, June-Sik Kim, Seungill Kim, Hye Yeon Soh, Hosub Shin, Hosung Jang, Ju Hyun Ryu, Ahyeong Kim, Kil-Young Yun, Shinje Kim, Ki Sun Kim, Doil Choi, Jin Hoe Huh

The data availability statement for this article is incorrect. The correct statement is: Data are available from the NCBI Short Read Archive (SRA) (http://www.ncbi.nlm.nih.gov/sra/?term=DRA001679).
