# Supplementary figures and images for: De Novo Transcriptome Analysis to Identify Anthocyanin Biosynthesis Genes Responsible for Tissue-Specific Pigmentation in Zoysiagrass (Zoysia japonica Steud.)
Source: PLoS One. 2015 Apr 23;10(4):e0124497. doi: 10.1371/journal.pone.0124497 (PMC4408010; doi:10.1371/journal.pone.0124497)

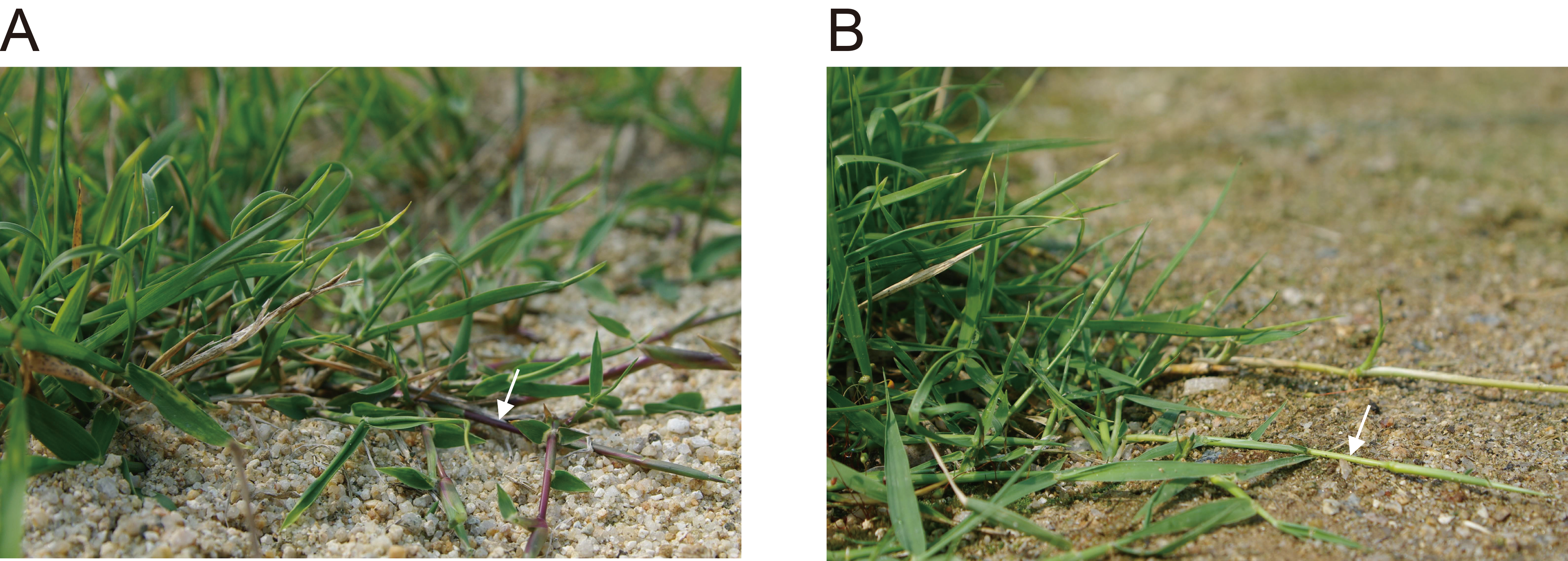

Supplement: S1 Fig — Anyang-jungji (A) and Greenzoa (B). Arrows indicate creeping stolons. (TIF) [file pone.0124497.s001.tif]

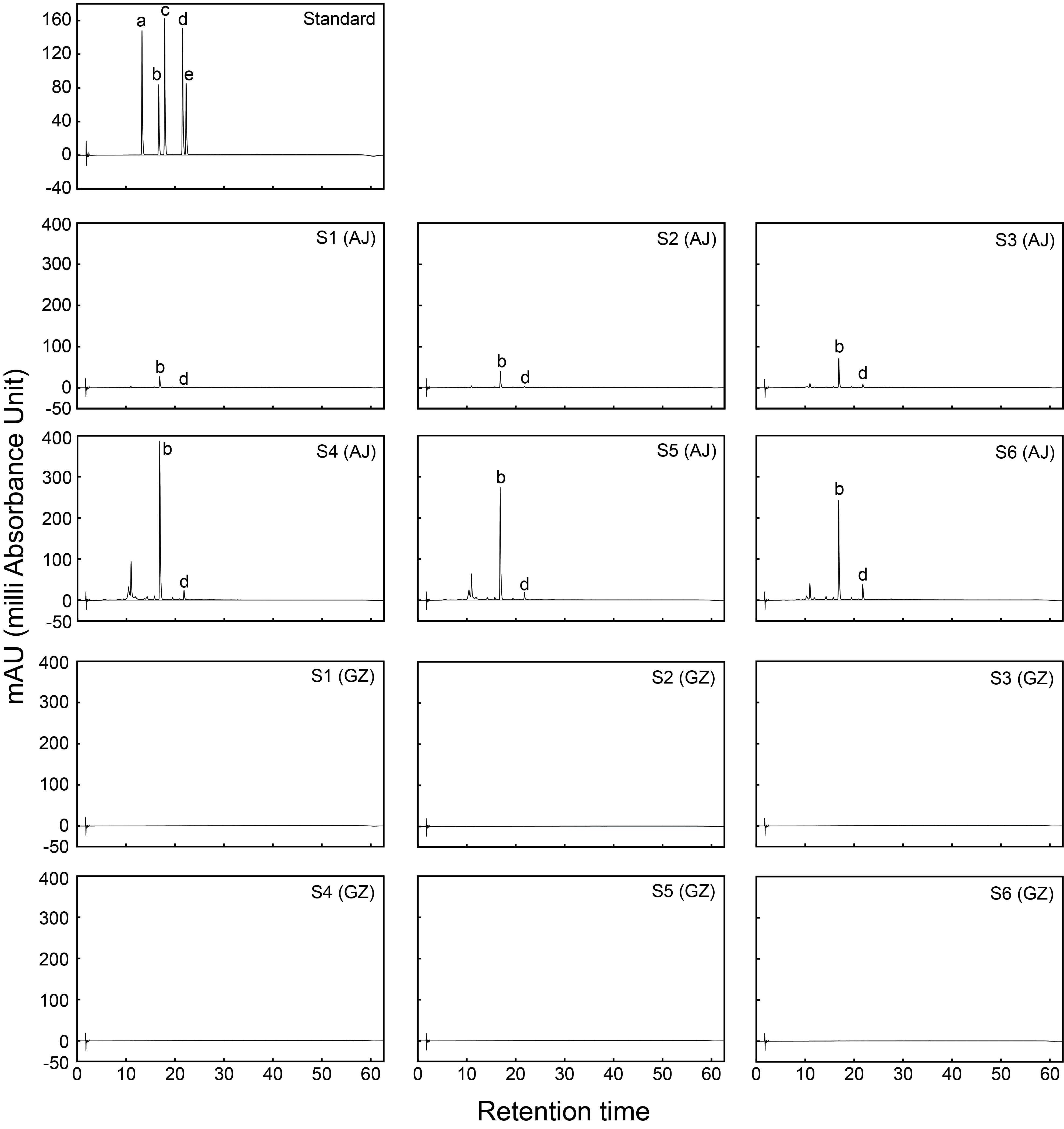

Supplement: S2 Fig — The chromatograms were recorded at 520 nm. a, delphinidin; b, cyanidin; c, peonidin; d, petunidin; e, malvidin. (TIF) [file pone.0124497.s002.tif]

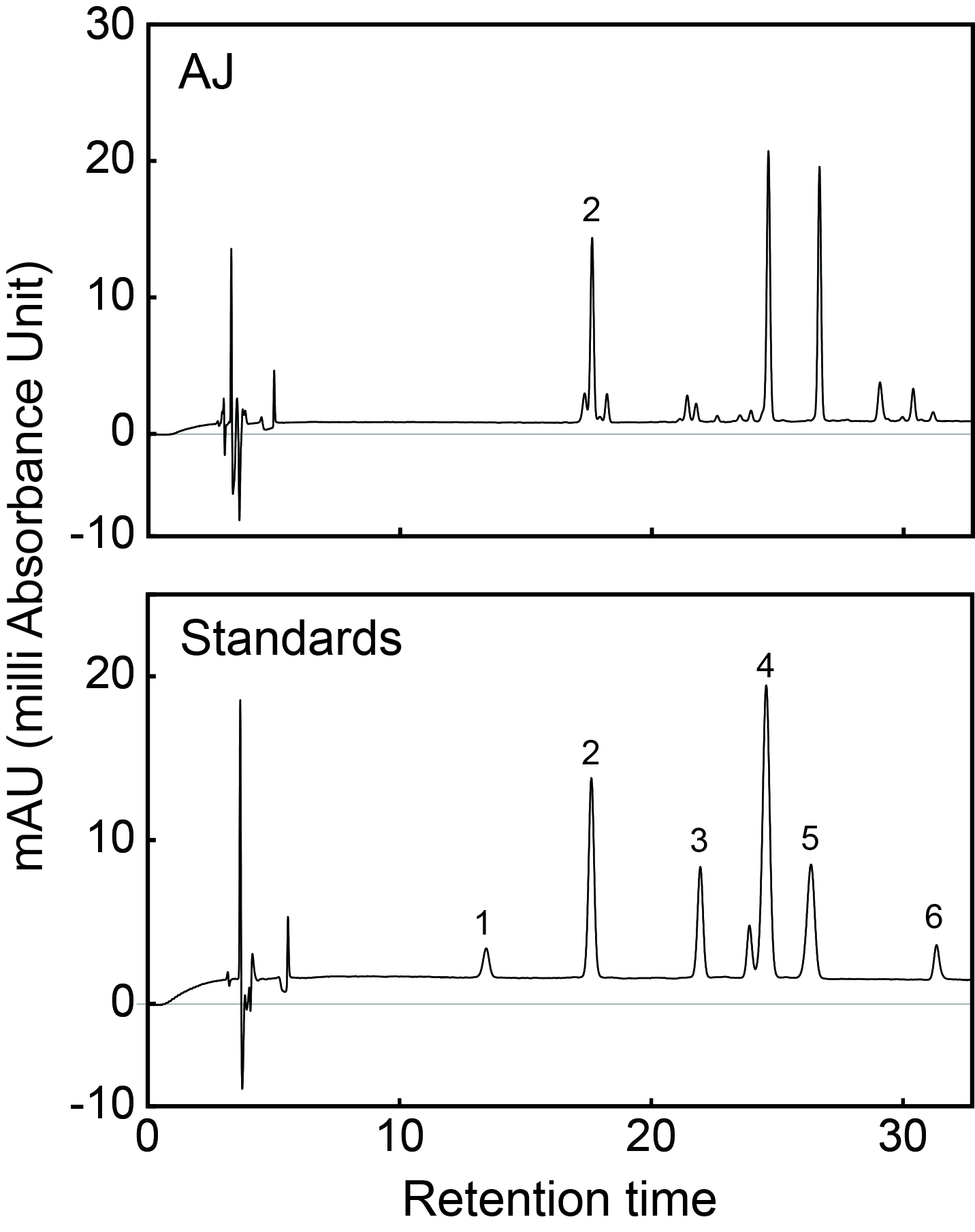

Supplement: S3 Fig — The chromatograms were recorded at 475 nm. 1, delphinidin-3-O-glucoside; 2, cyanidin-3-O-glucoside; 3, pelargonidin-3-O-glucoside; 4, peonidin-3-O-glucoside; 5, malvidin-3-O-glucoside; 6, luteolinidin. (TIF) [file pone.0124497.s003.tif]

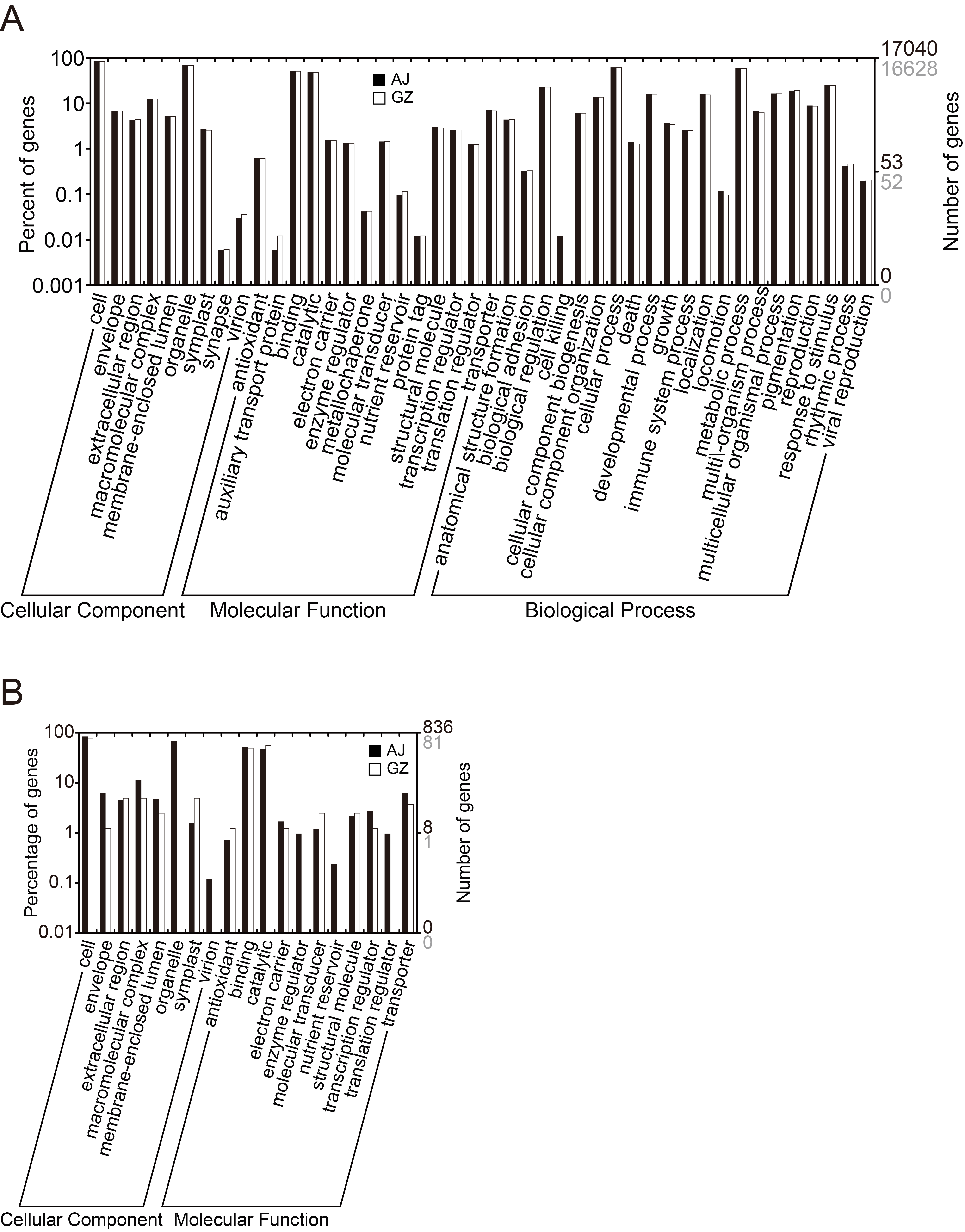

Supplement: S4 Fig — Gene ontology (GO) terms for each zoysiagrass transcripts were assigned based on significant TBLASTX hits (e-value < 1e-15) against the NR database. (A) The results are summarized in three main categories (biological process, molecular function, and cellular component) and 44 subcategories. (B) Gene ontology classification of DEGs between AJ and GZ. (TIF) [file pone.0124497.s004.tif]

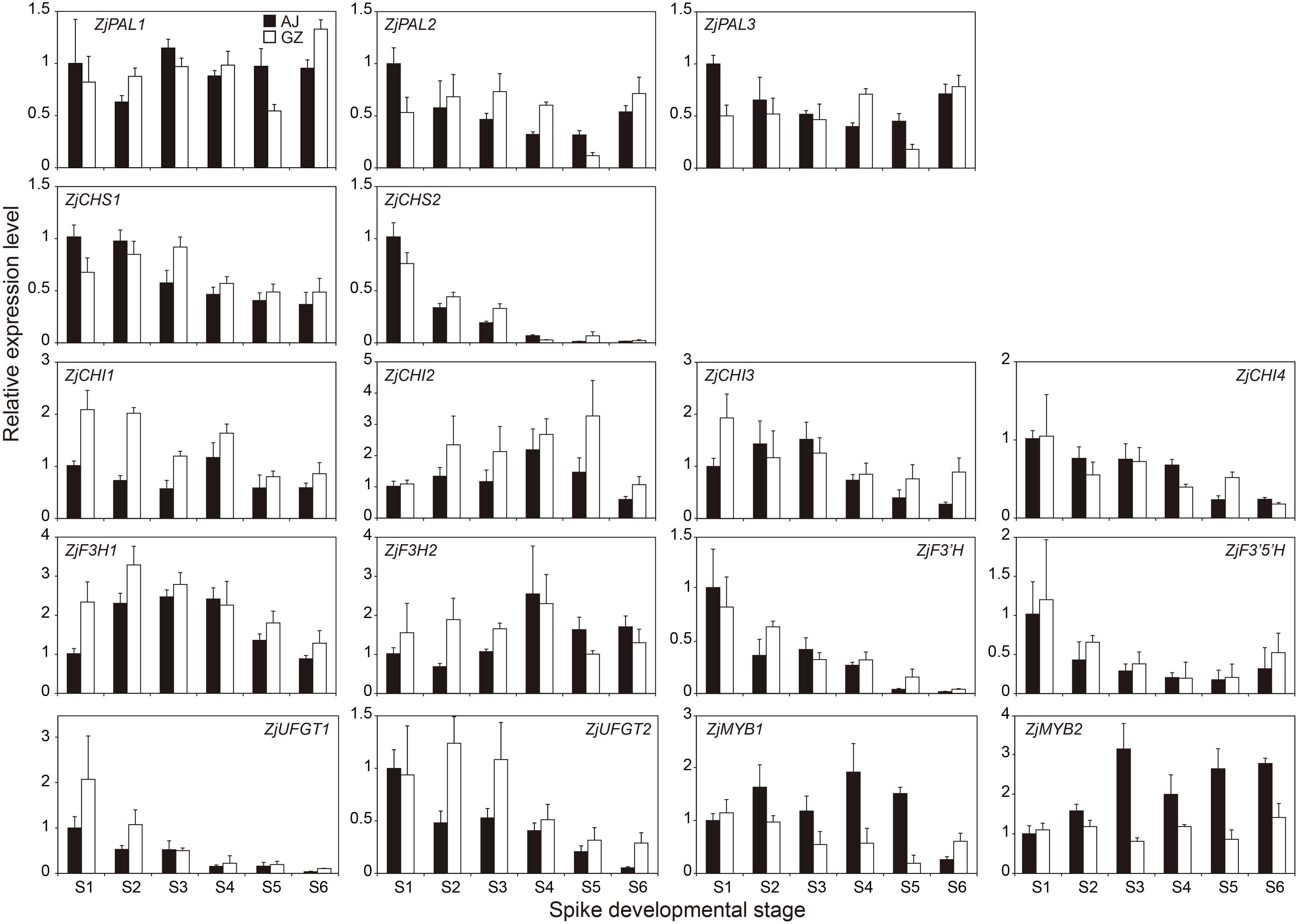

Supplement: S5 Fig — The relative expression level of each transcript was determined by qRT-PCR. All values are normalized relative to the mean abundance of β-ACTIN at each stage. Bars represent means ± SD from triplicate biological repeats. (TIF) [file pone.0124497.s005.tif]

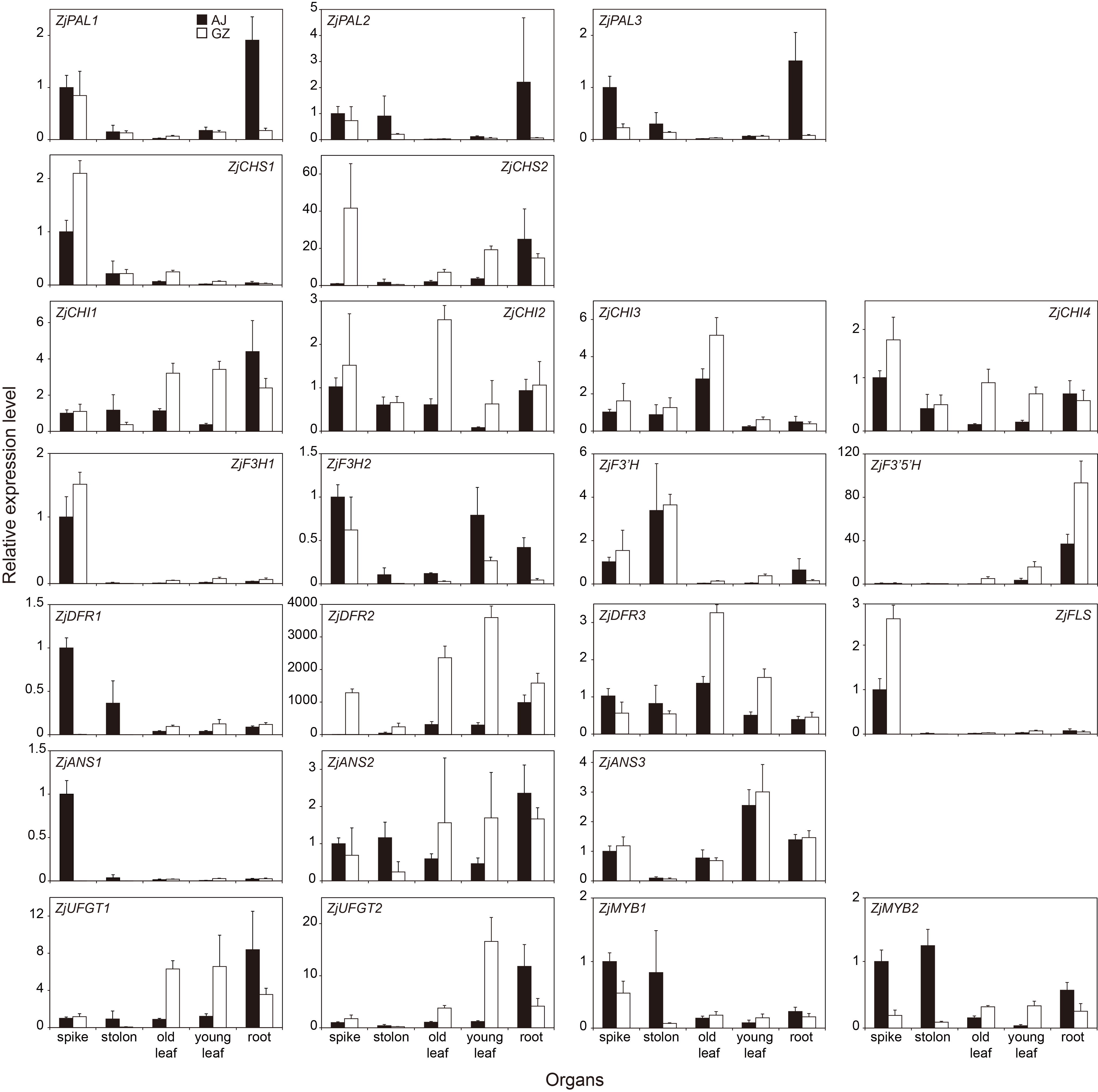

Supplement: S6 Fig — The relative expression level of each transcript was determined by qRT-PCR. All values are normalized relative to the mean abundance of β-ACTIN. Bars represent means ± SD from triplicate biological repeats. (TIF) [file pone.0124497.s006.tif]

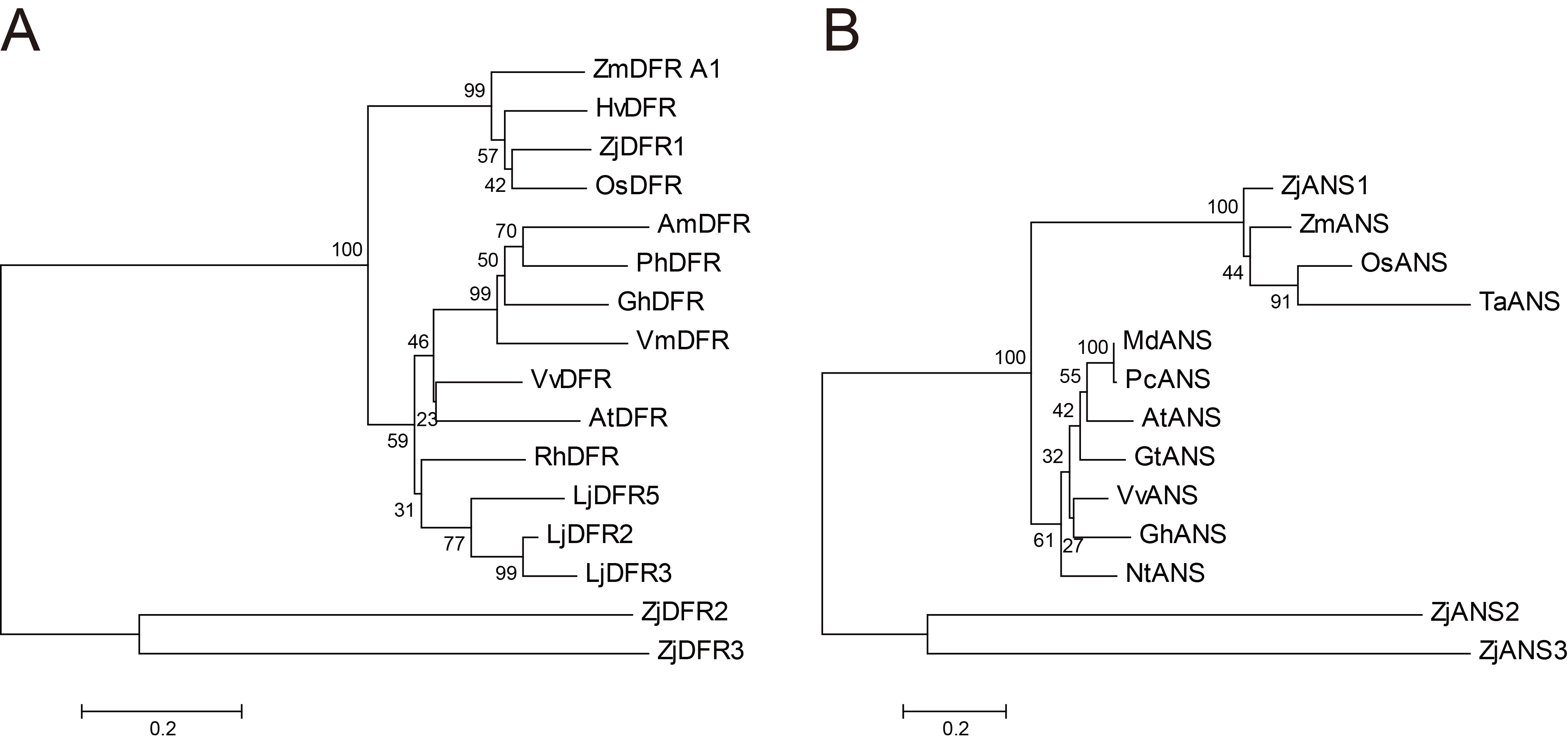

Supplement: S7 Fig — (A) Phylogeny of the DFR protein family. (B) Phylogeny of the ANS protein family. Am, Antirrhinum majus, At, Arabidopsis thaliana; Gh, Gerbera hybrid; Gt, Gentiana triflora; Hv, Hordeum vulgare; Lj, Lotus japonicus; Md, Malus domestica; Nt, Nicotiana tabacum; Os, Oryza saiva; Pc, Pyrus communis; Ph, Petunia hybrid; Rh, Rosa hybrid; Ta, Triticum aestivum; Vm, Vaccinium macrocarpon; Vv, Vitis vinifera; Zm, Zea mays. Accession numbers of the proteins analyzed are listed in the S8 Table. (TIF) [file pone.0124497.s007.tif]

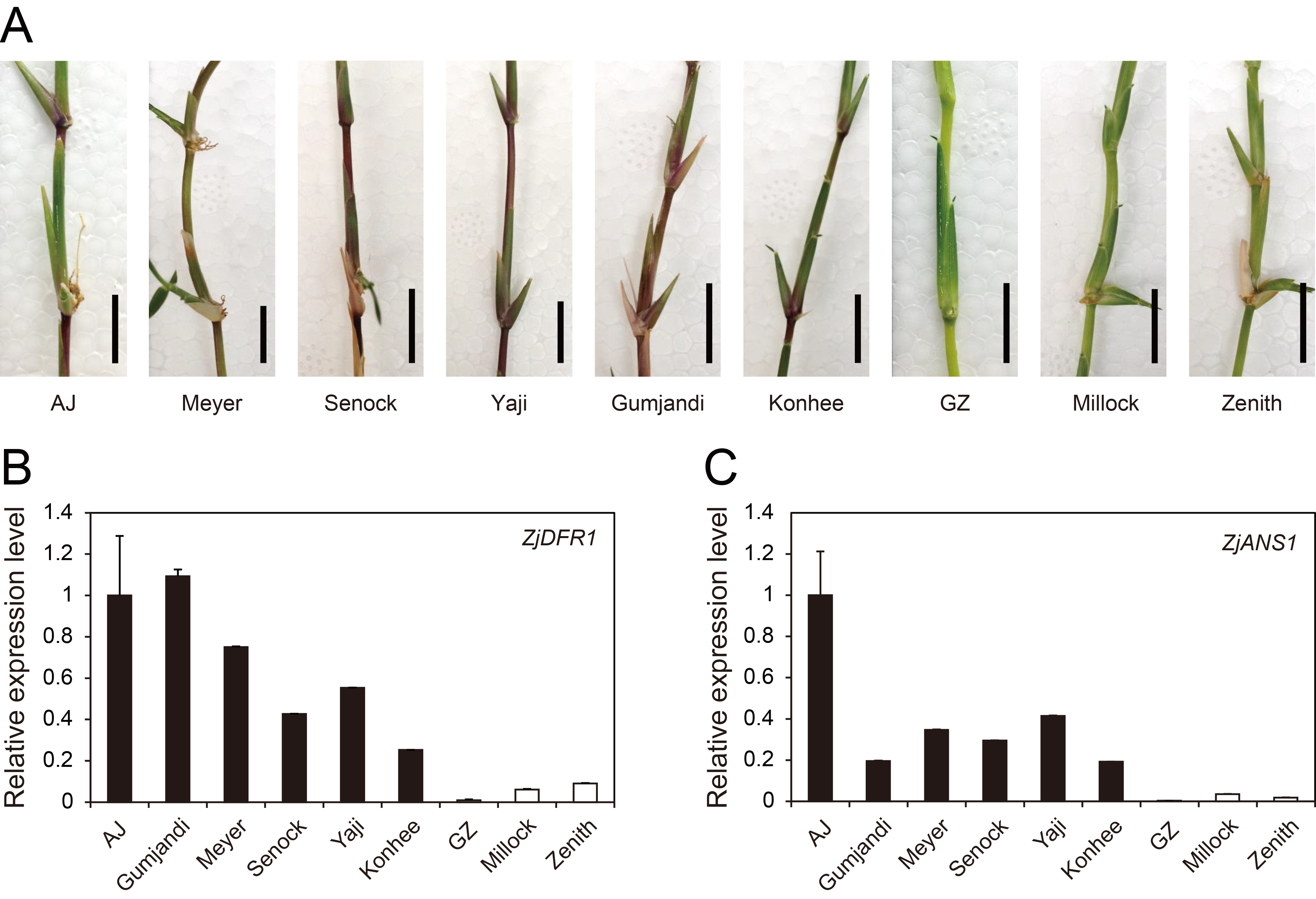

Supplement: S8 Fig — (A) Representative pictures of the stolon of nine zoysiagrass cultivars. The guide bar indicates 10 mm. (B and C) Expression patterns of ZjDFR1 (B) and ZjANS1 (C) at stolon by qRT-PCR. All values are normalized relative to the mean abundance of β-ACTIN. Bars represent means ± SD from triplicate biological repeats. (TIF) [file pone.0124497.s008.tif]

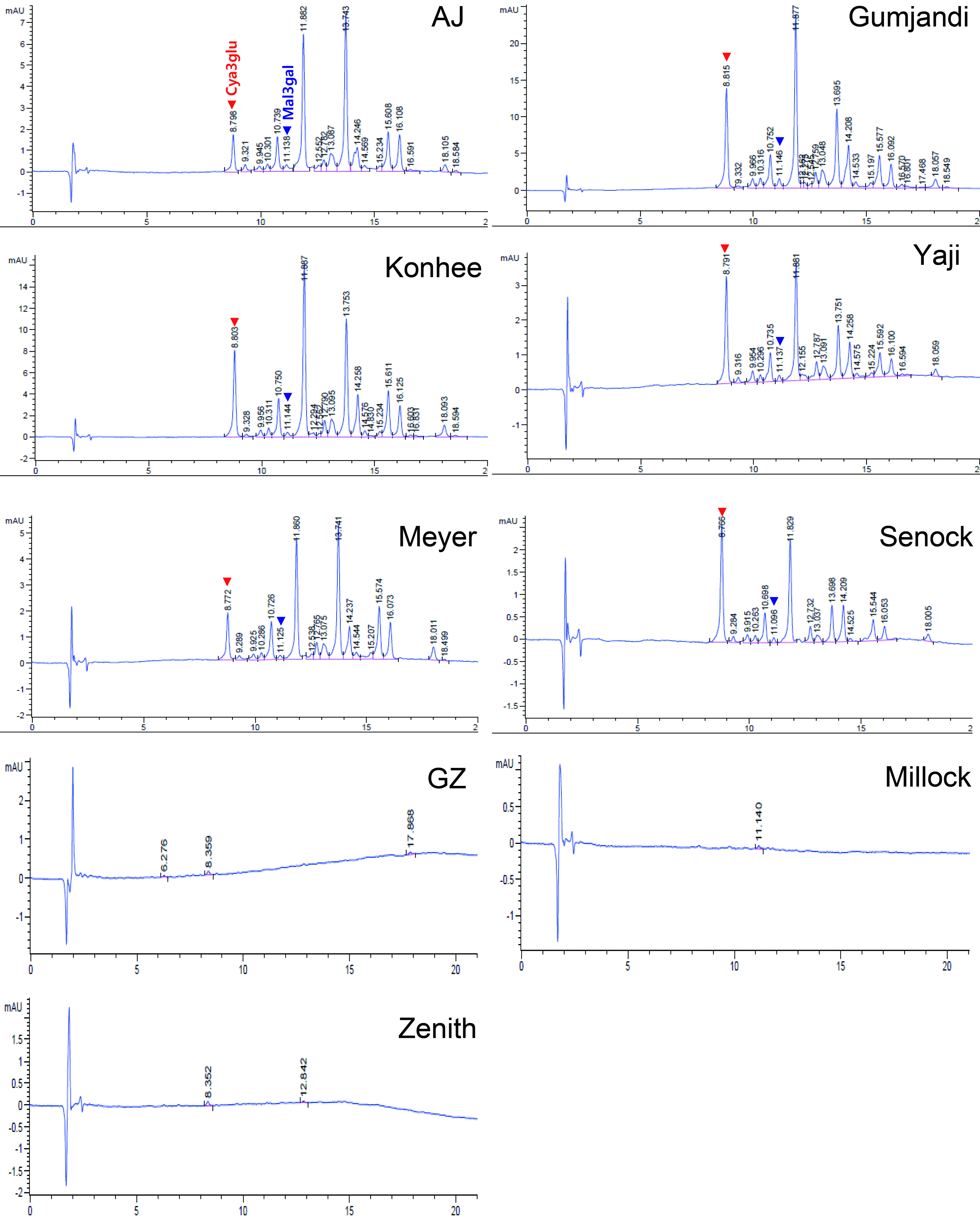

Supplement: S9 Fig — The chromatograms were recorded at 520 nm. Red and blue arrowheads indicate cyanidin-3-O-glucoside and malvidin-3-O-glucoside, respectively. (TIF) [file pone.0124497.s009.tif]

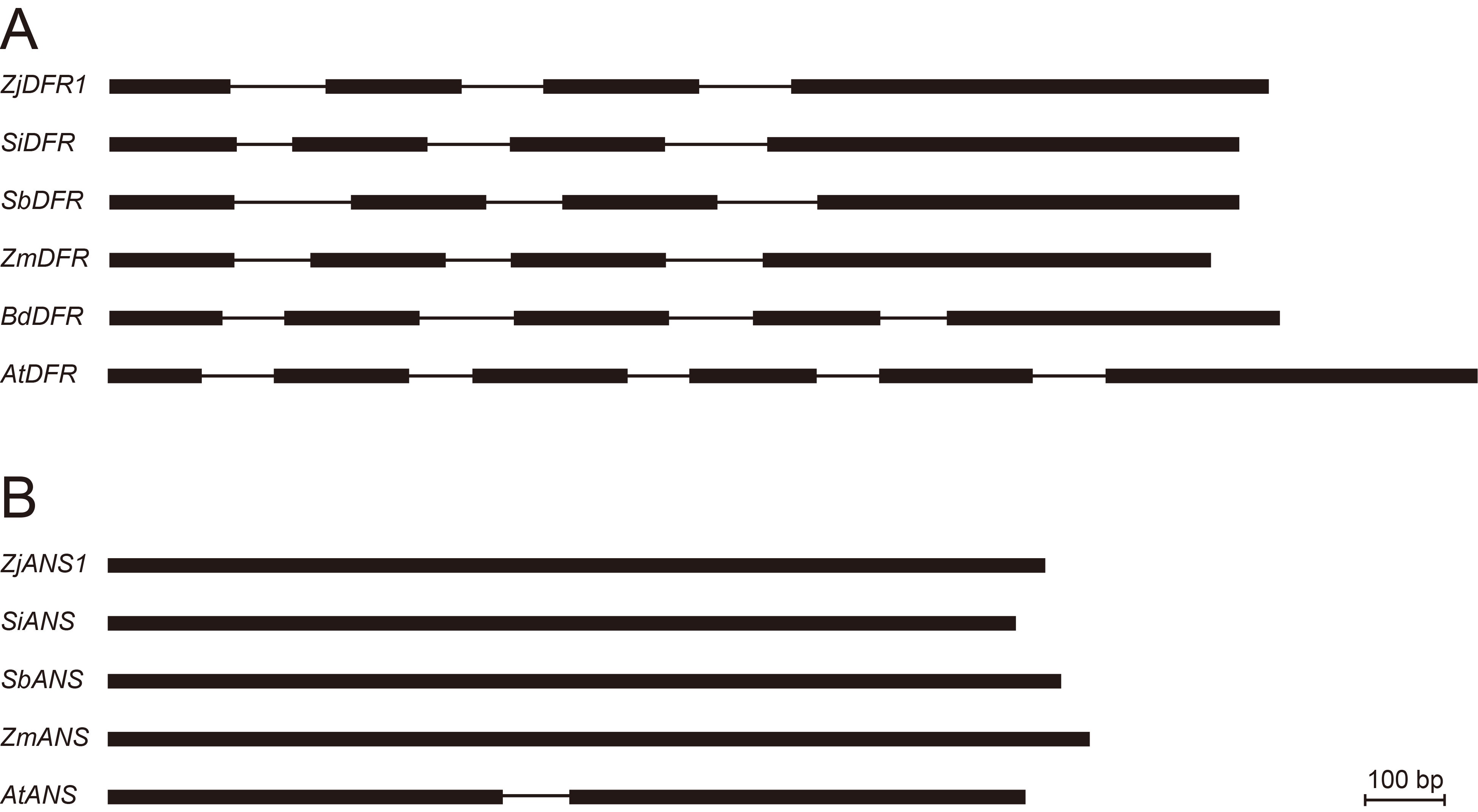

Supplement: S10 Fig — Closed bars and lines represent exons and introns, respectively. Zj, Zoysia japonica; Si, Setaria italica; Sb, Sorghum bicolor; Zm, Zea mays; Bd, Brachypodium distachyon; At, Arabidopsis thaliana. (TIF) [file pone.0124497.s010.tif]

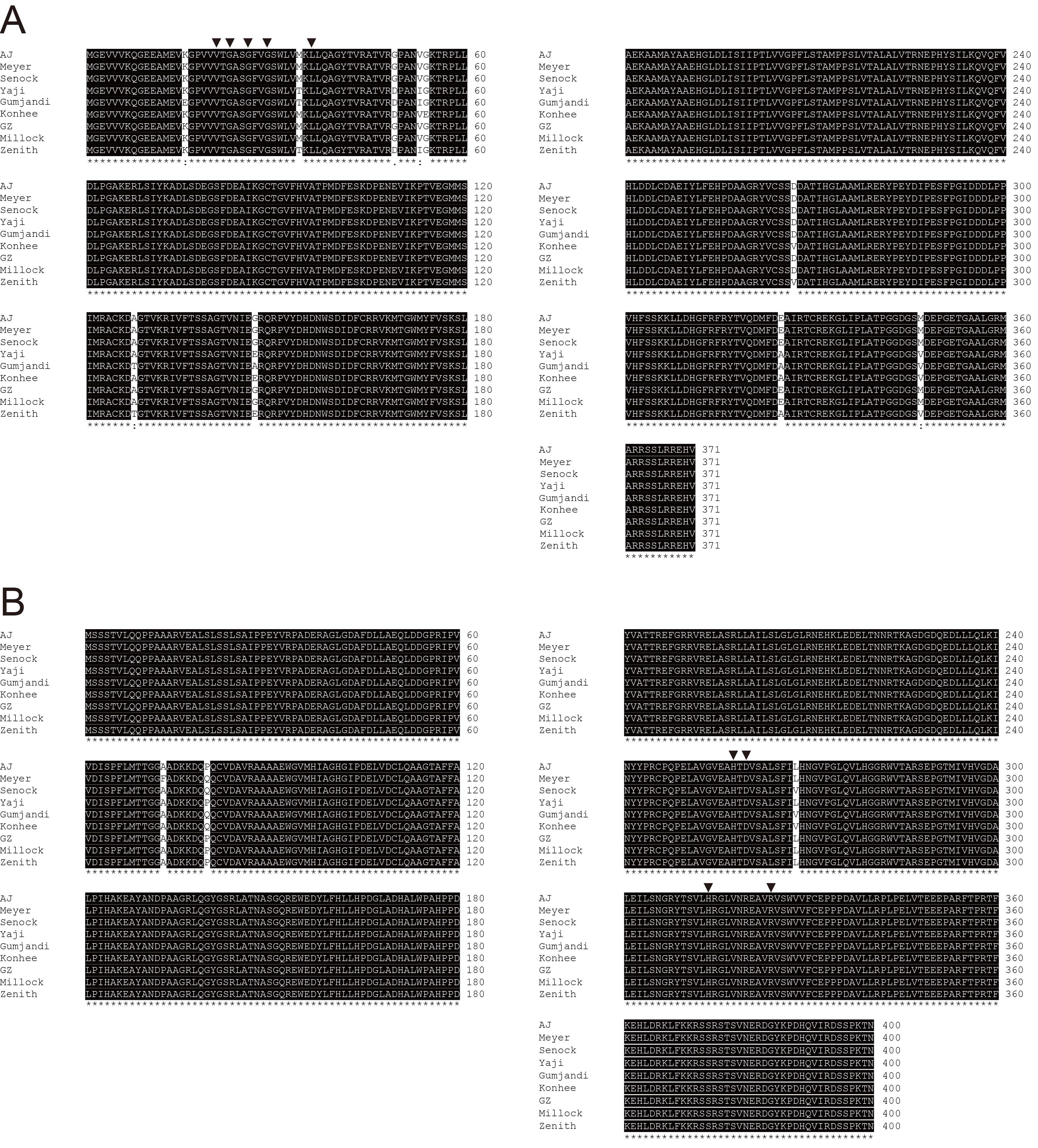

Supplement: S11 Fig — (A) Arrowheads on ZjDFR1 indicate conserved amino acid residues in the hydroxysteroid dehydrogenase/DFR superfamily [51]. (B) Arrowheads on ZjANS1 indicate conserved His and Asp residues required for ferrous-iron coordination, and Arg for putative 2-oxoglutarate binding site [52]. (TIF) [file pone.0124497.s011.tif]

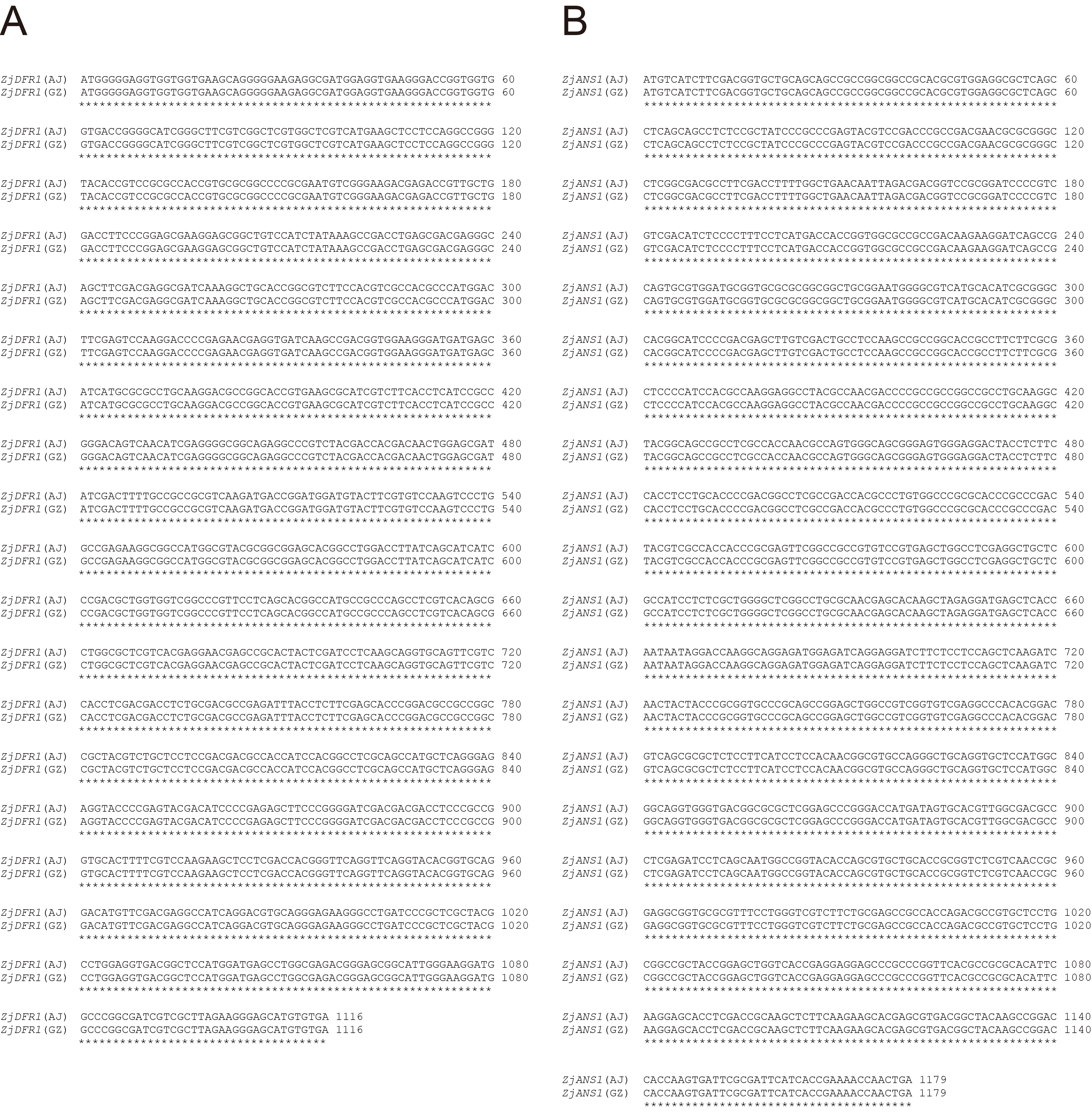

Supplement: S12 Fig — (TIF) [file pone.0124497.s012.tif]

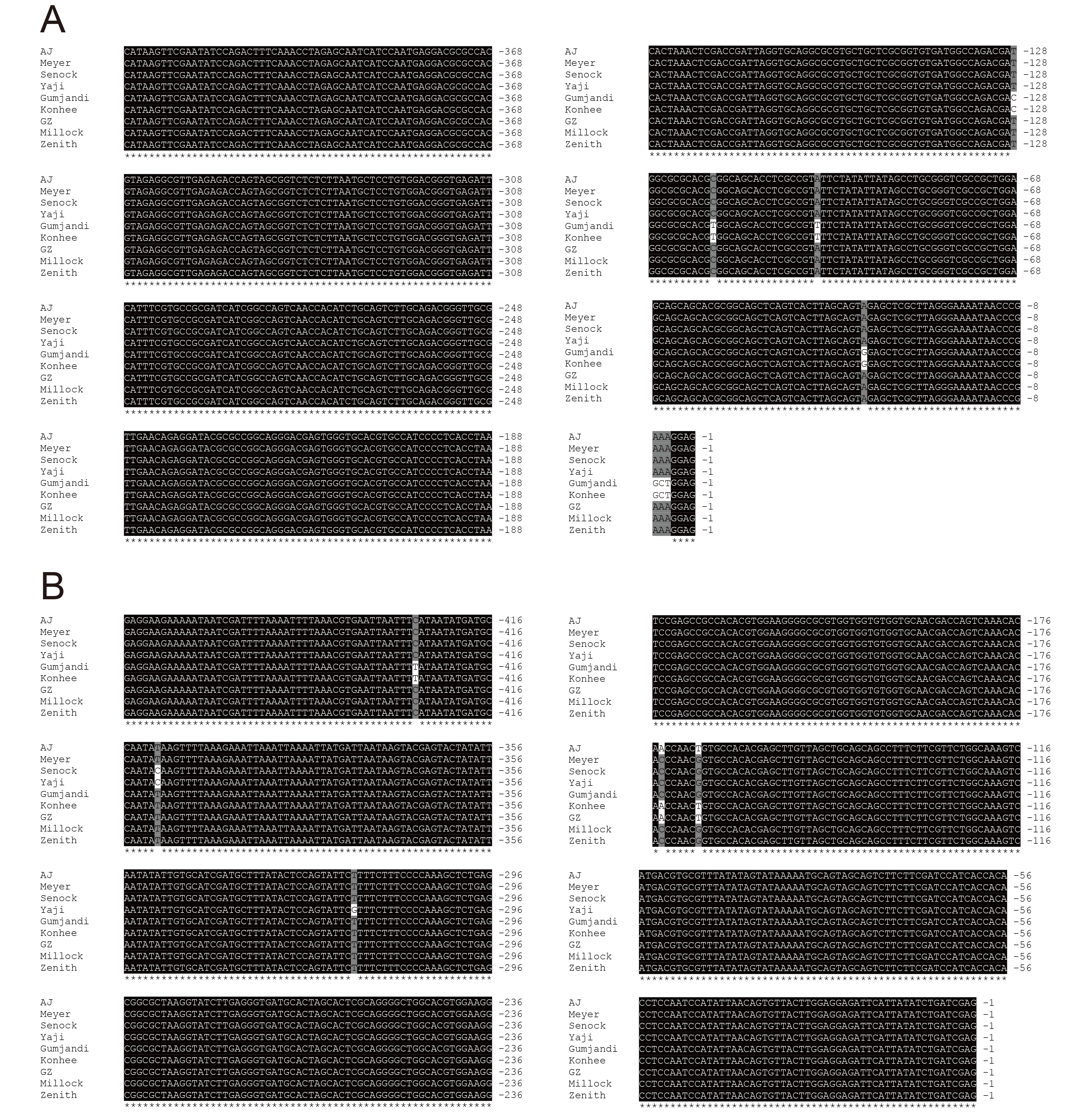

Supplement: S13 Fig — (TIF) [file pone.0124497.s013.tif]

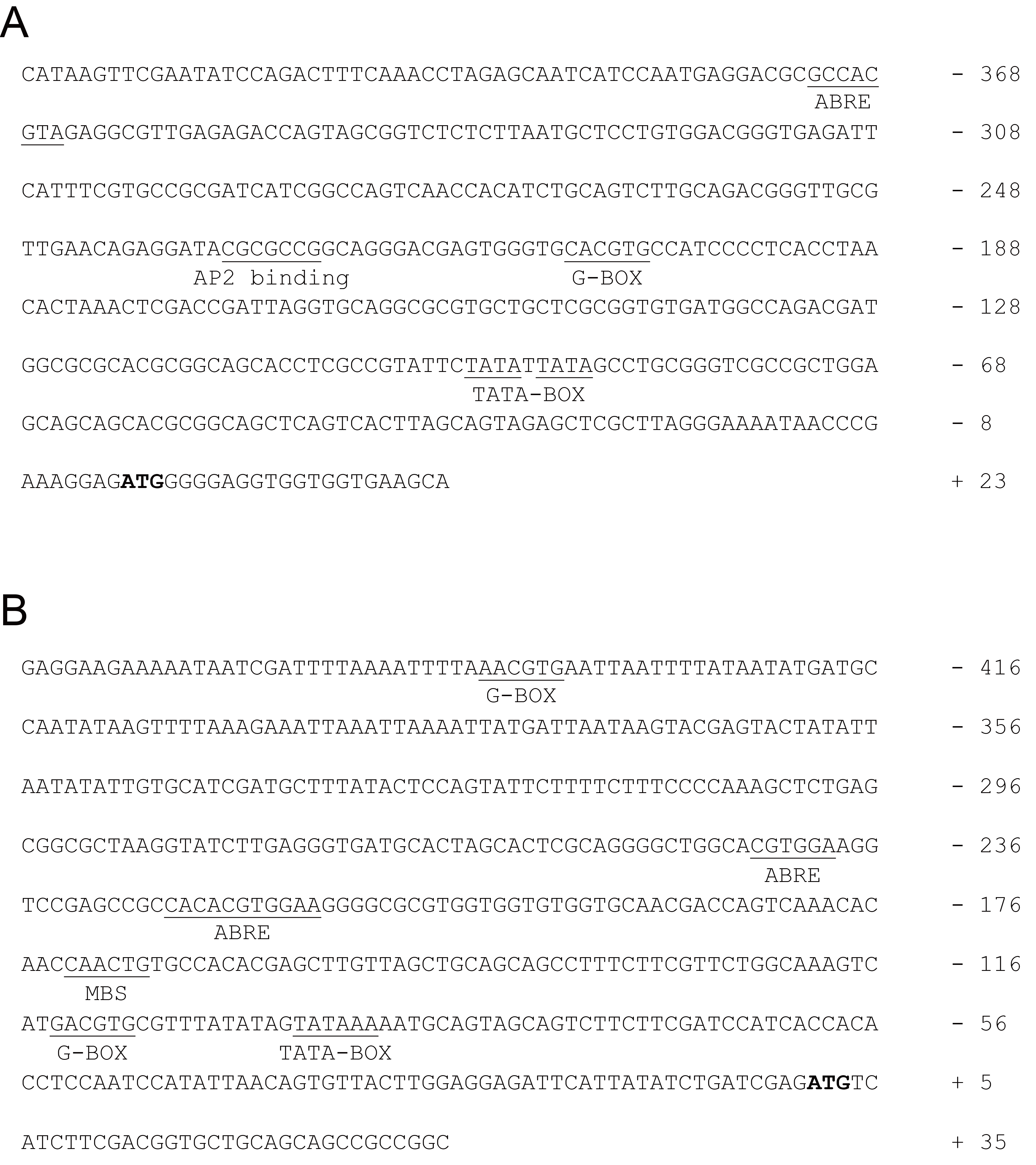

Supplement: S14 Fig — The bold ‘ATG’ indicates the translational start site. Predicted cis-regulatory elements are underlined and identified according to the PlantCare database [49]. ABRE, abscisic acid response element; MBS, MYB binding site; AP2, Apetala2. (TIF) [file pone.0124497.s014.tif]

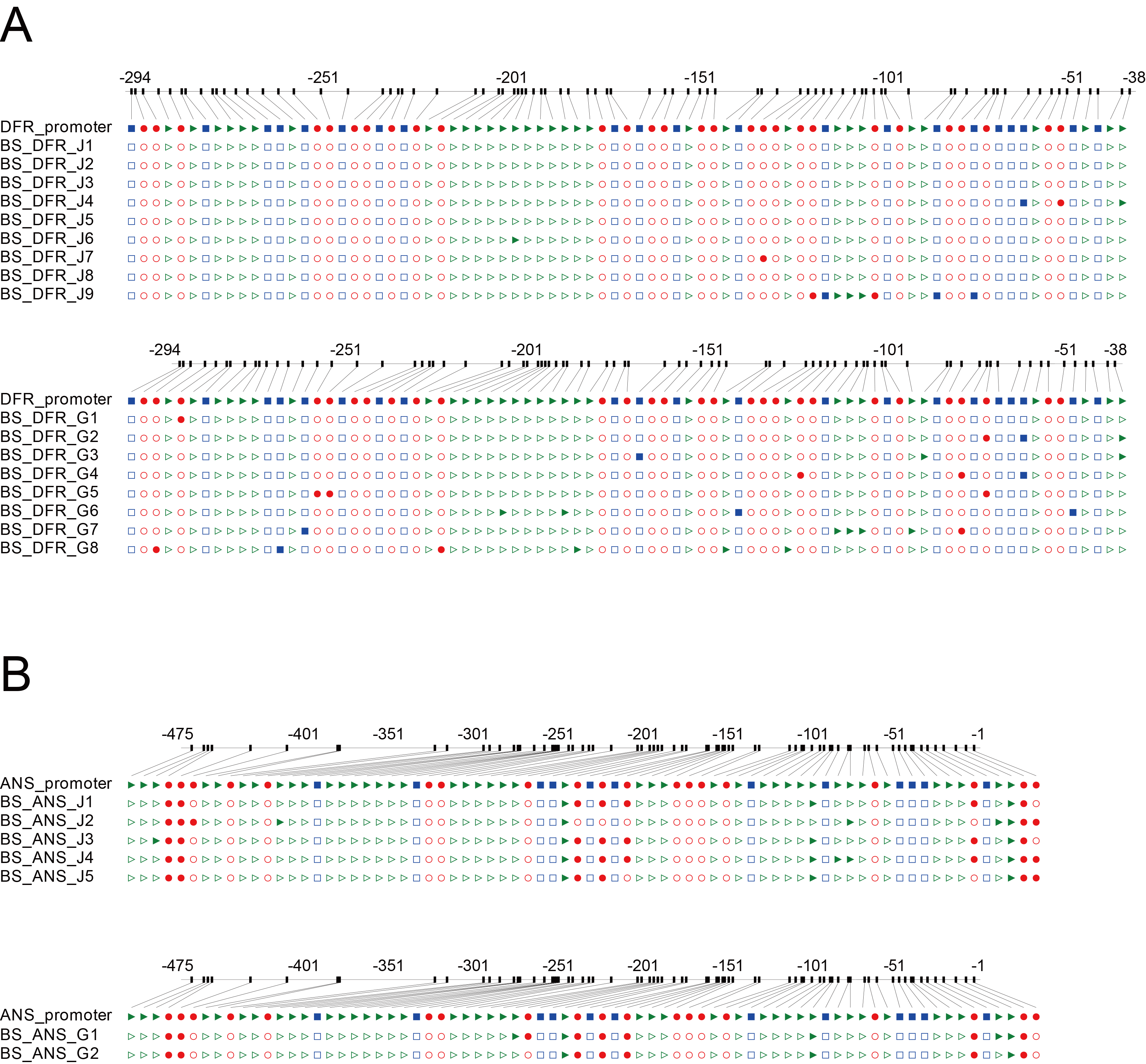

Supplement: S15 Fig — Numbers are from the translational start site. 5-methylcytosines in the CG (circle), CHG (triangle), and CHH (square) contexts were displayed by CyMATE [53]. Open and closed shapes indicate unmethylated and methylated cytosines, respectively. Primers used for bisulfite sequencing are listed in S12 Table. (TIF) [file pone.0124497.s015.tif]

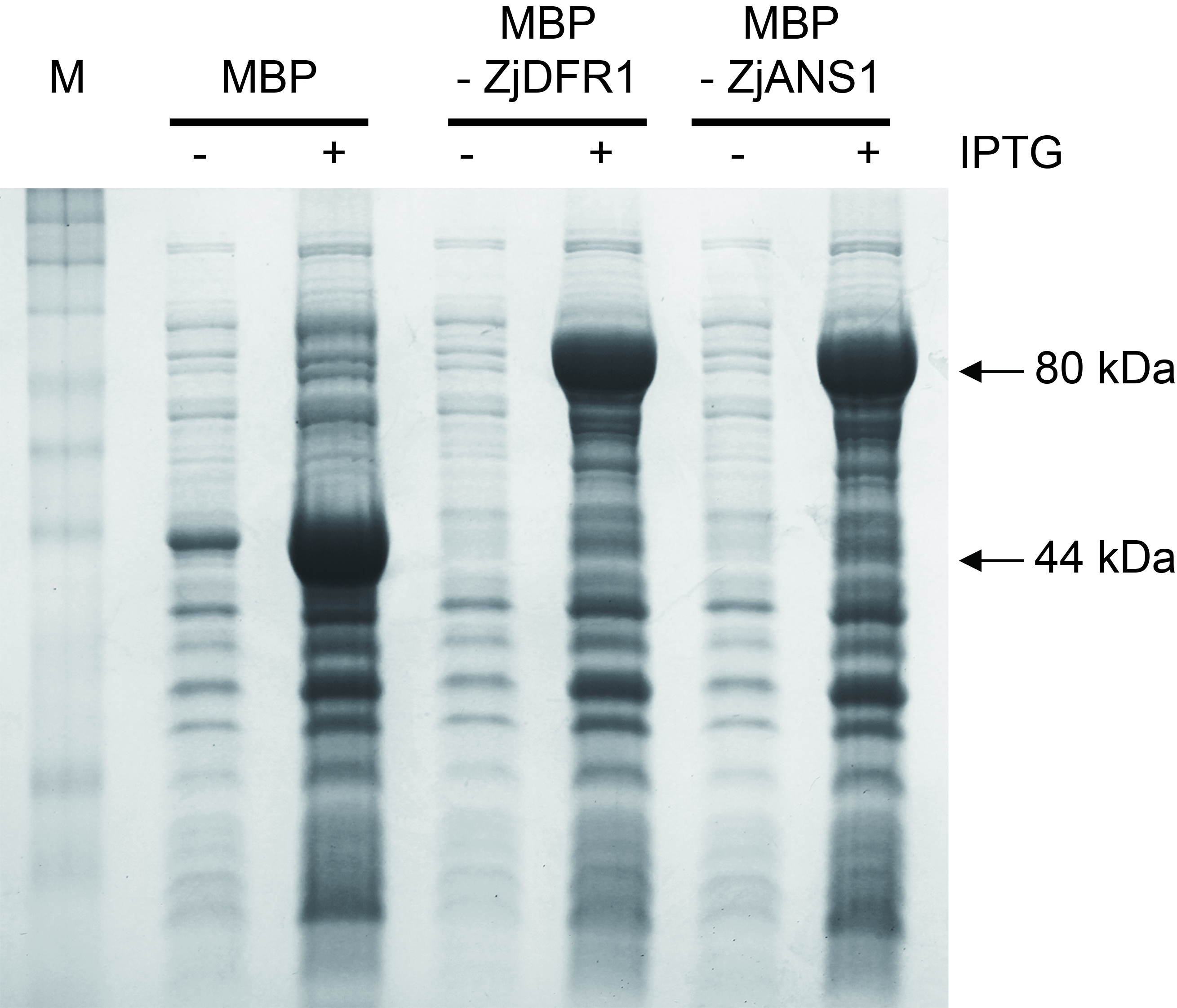

Supplement: S16 Fig — Total proteins were separated on a 10% SDS-PAGE gel and visualized by Coomassie Brilliant Blue staining. Expected molecular weights of MBP, MBP-ZjDFR1, and MBP-ZjANS1 are indicated to the right side of the panel. M, size marker. (TIF) [file pone.0124497.s016.tif]

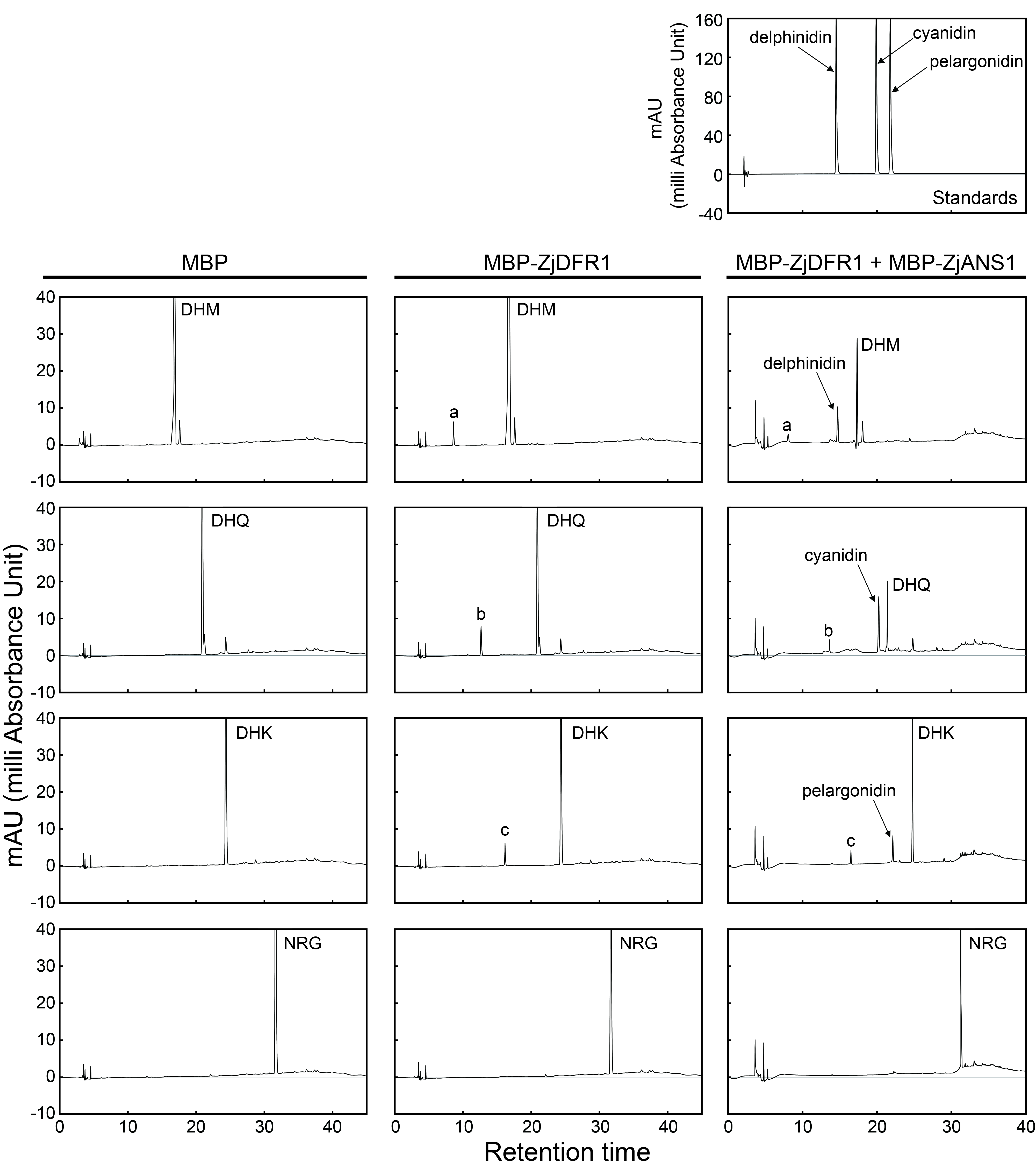

Supplement: S17 Fig — The chromatograms were recorded at 280 nm. a, leucodelphinidin; b, leucocyanidin; c, leucopelargonidin. (TIF) [file pone.0124497.s017.tif]

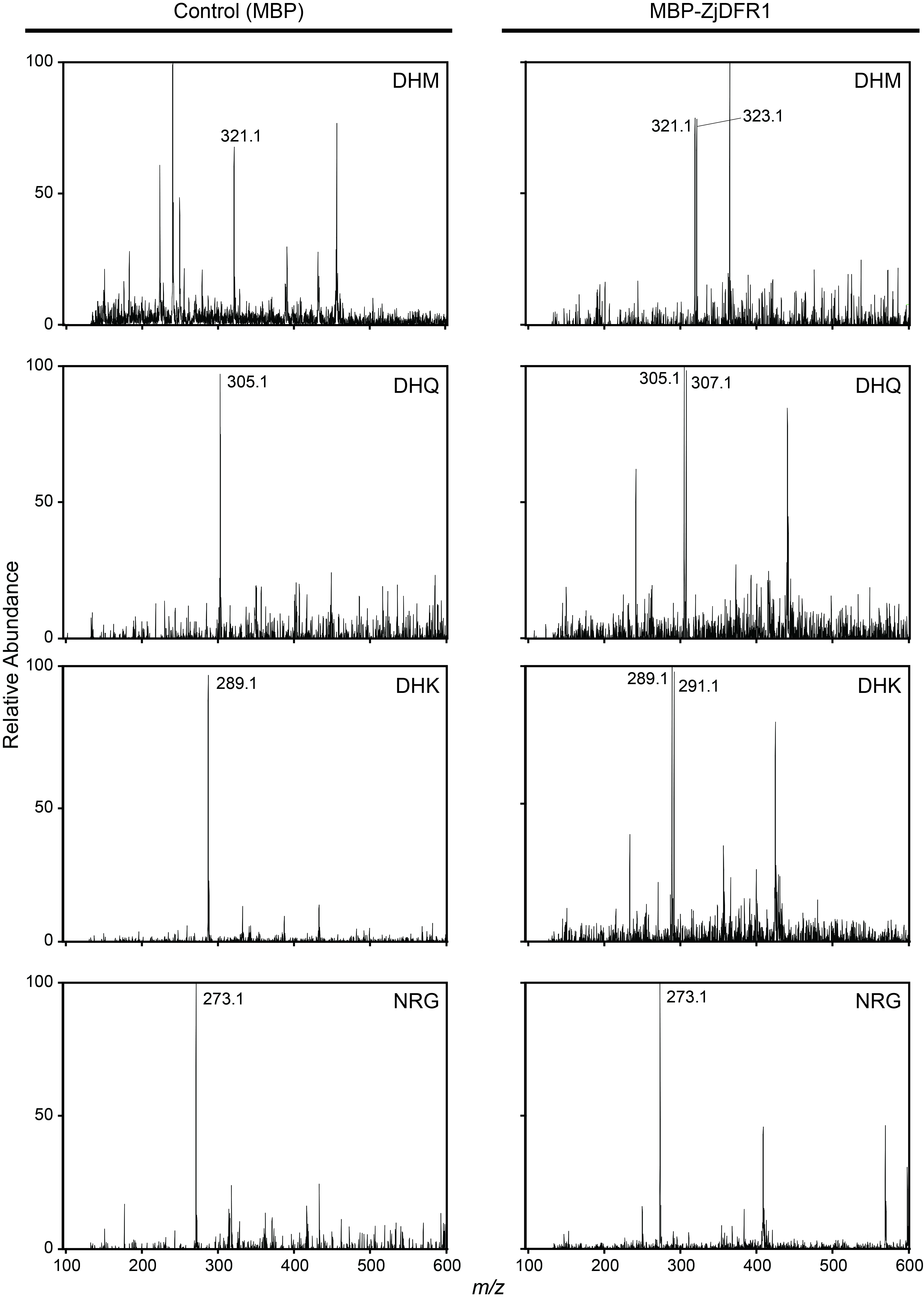

Supplement: S18 Fig — Mass spectra obtained for bacterial cell extracts incubated with four substrates and the [M+] (m/z) values of the corresponding peaks: dihydromyricetin (DHM; m/z = 321), dihydroquercetin (DHQ; m/z = 305), dihydrokaempferol (DHK; m/z = 289), and naringenin (NRG; m/z = 273). (TIF) [file pone.0124497.s018.tif]

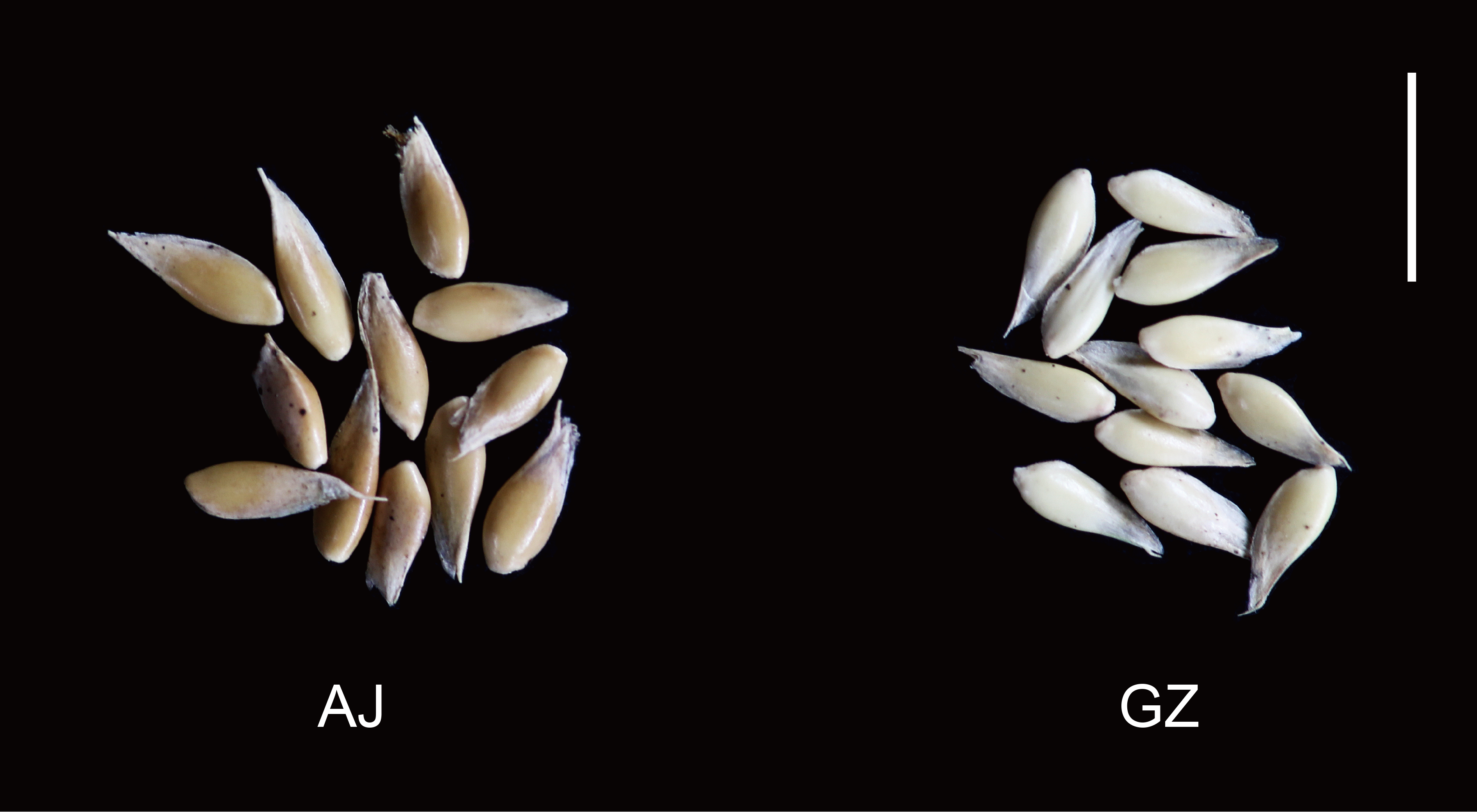

Supplement: S19 Fig — The guide bar indicates 0.5 mm. (TIF) [file pone.0124497.s019.tif]
