# Supplementary material for: De Novo Transcriptome Analysis to Identify Anthocyanin Biosynthesis Genes Responsible for Tissue-Specific Pigmentation in Zoysiagrass (Zoysia japonica Steud.)
Source: PLoS One. 2015 Apr 23;10(4):e0124497. doi: 10.1371/journal.pone.0124497 (PMC4408010; doi:10.1371/journal.pone.0124497)
Supplement: S1 File — (DOC) [file pone.0124497.s020.doc]

File S1. Alignment of ZjDFR1 and ZjANS2 homologs

A. Alignment of ZjDFR1 homologs

ZjDFR1 VVVTGASGFVGSWLVMKLLQAGYTVRATVRGPANVGKTRPLLDLPGAKERLSIYKADLSD

ZmDFR VLVTGASGFVGSWLVMKLLQAGYTVRATVRDPANVGKTKPLMDLXGATERLSIWKADLAE

HvDFR VVVTGASGFVGSWLVMKLLQAGYTVRATVRDPANVEKTKPLLELPGAKERLSIWKADLSE

OsDFR VVVTGASGFVGSWLVMKLLQAGYTVRATVRDPSNVGKTKPLLELAGSKERLTLWKADLGE

AmDFR VCVTGAAGFIGSWLVMRLLERGYTVRATVRDPGNMKKVKHLIELPKADTNLTLWKADMTV

GhDFR VCVTGAAGFIGSWLVMRLLERGYVVHATVRDPGDLKKVKHLLELPKAQTNLKLWKADLTQ

VmDFR VCVTGAAGFIGSWLIMRLLERGYVVRATVRDPGNLKKVKHLLELPKADTNLTLWKADLNE

PhDFR VCVTGAAGFIGSWLVMRLLERGYNVHATVRDPENKKKVKHLLELPKADTNLTLWKADLTV

LjDFR2 VCVTGAAGFIGSWLVMRLMERGYMVRATVRDPANMKKVKHLLELPEAKTKLTLWKADLAE

LjDFR3 VCVTGSTGFIGSWLVMRLMEGGYTVRATVRDPDNMKKVKHLLELPGAKTNLTIWNADLTE

LjDFR5 VCVTGAAGFIGSWLVMRLIERGYTVRATIRDPANMKKVKHLLELPDAKTKLSLWKADLAE

RhDFR VCVTGASGFIGSWLVMRLLDRGYTVRATVRDPANKKKVNHLLDLPKAATHLTLWKADLAE

VvDFR VCVTGASGFIGSWLVMRLLERGYTVRATVRDPTNVKKVKHLLDLPKAETHLTLWKADLAD

AtDFR VCVTGASGFIGSWLVMRLLERGYFVRATVRDPGNLKKVQHLLDLPNAKTLLTLWKADLSE

ZjDFR2 VCVTGASGFLASWLIRRLLEAGYHVRGTVRDPGNRQKVAHLWKLPGAKERLQIVRADLLE

ZjDFR3 -CVTGASGYIASWITKLLLDRGYTVRATVRDTADPKKTQHLRDLDGAKDRLHLFKASLLD

ZjDFR1 EGSFDEAIKGCTGVFHVATPMDFESKDPENEVIKPTVEGMMSIMRACKDAGTVKRIVFTS

ZmDFR EGSFHDAIRGCTGVFHVATPMDFLSKDPENEVIKPTVEGMISIMRACKEAGTVRRIVFTS

HvDFR DGSFNEAIAGCTGVFHVATPMDFDSQDPENEVIKPTVEGMLSIMRACKEAGTVKRIVFTS

OsDFR EGSFDAAIRGCTGVFHVATPMDFESEDPENEVVKPTVEGMLSIMRACRDAGTVKRIVFTS

AmDFR EGSFDEAIQGCEGVFHLATSMEFDSVDPENEVIKPTIDGMLNIIKSCVQAKTVKKFIFTT

GhDFR EGSFDEAIQGCHGVFHLATPMDFESKDPENEIIKPTIEGVLSIIRSCVKAKTVKKLVFTS

VmDFR EGSFDEAIEGCVGVFHVATPMDFESKDPENEVIKPTINGVLSIIKSCTKAKTVKRLVFTS

PhDFR EGSFDEAIQGCQGVFHVATPMDFESKDPENEVIKPTVRGMLSIIESCAKANTVKRLVFTS

LjDFR2 EGSFDEAIKGCTGVFHVATPMDFESKDPENEVIKPTINGVLDIMKACQKAKTVRRLVFTS

LjDFR3 EGSFDEAINGCSGVFHVASPMDFNSKDPENEVIKPSINGVLDIMKACQKAKTVRRLVFTS

LjDFR5 EGSFDEAIRGCTGVFHVATPMDFESKDPENEVIKPTINGLLDILKACEKAKTVRRLVFTS

RhDFR EGSFDEAIKGCTGVFHVATPMDFESKDPENEVIKPTINGVLDIMQACLKAKTVRRLVFTS

VvDFR EGSFDEAIKGCTGVFHVATPMDFESKDPENEVIKPTIEGMLGIMKSCAAAKTVRRLVFTS

AtDFR EGSYDDAINGCDGVFHVATPMDFESKDPENEVIKPTVNGMLGIMKACVKAKTVRRFVFTS

ZjDFR2 EGSFDDAVMACEGVFHTASPVLANCDSSKEETLVPAVHGTLNVLRSCKKNPFLKRVVLTS

ZjDFR3 EGSFDDAVNGCDCVFHTASPFYHNVKDPKAELIDPAVKGTLNVLSSCKK-ASIKRVVVTS

ZjDFR1 SAGTVNIEGRQRPVYDHDNWSDIDFCRRVKMTGWMYFVSKSLAEKAAMAYAAEHGLDLIS

ZmDFR SAGTVNLEERQRPVYDEESWTDVDFCRRVKMTGWMYFVSKTLAEKAALAYAAEHGLDLVT

HvDFR SAGSVNIEERPRPAYDQDNWSDIDYCRRVKMTGWMYFVSKALAEKAAMEYASENGLDFIS

OsDFR SAGTVNIEERQRPSYDHDDWSDIDFCRRVKMTGWMYFVSKSLAEKAAMEYAREHGLDLIS

AmDFR SGGTVNVEEHQKPVYDETDSSDMDFINSKKMTGWMYFVSKILAEKAGMEAAKENNIDFIS

GhDFR SAGTVNGQEKQLHVYDESHWSDLDFIYSKKMTAWMYFVSKTLAEKAAWDATKGNNISFIS

VmDFR SAGAVVDQEHQPLVFDENNWSDVDFLYDKKMTGWTYFVSKTLAERAAMEAAKEISIDFIS

PhDFR SAGTLDVQEQQKLFYDQTSWSDLDFIYAKKMTGWMYFASKILAEKAAMEEAKKKNIDFIS

LjDFR2 SAGTLNVIEHQKQMFDESCWSDVEFCRRVKMTGWMYFVSKTLAEQEAWKFAKEHGIDFIT

LjDFR3 SAGTLNAVEHQKQMCDESCWSDVEFCRRVKMTGWMYFVSKTLAEQEAWKFAQEHDIDFIT

LjDFR5 SAGTVDVTEHPKPVIDETCWSDIEFCLRVKMTGWMYFVSKTRAEQEAWKYAKEHNIDFVS

RhDFR SAGSVNVEETQKPVYNESNWSDVEFCRRVKMTGWMYFASKTLAEQEAWKFAKENNIDFIT

VvDFR SAGTVNIQEHQLPVYDESCWSDMEFCRAKKMTAWMYFVSKTLAEQAAWKYAKENNIDFIT

AtDFR SAGTVNVEEHQKNVYDENDWSDLEFIMSKKMTGWMYFVSKTLAEKAAWDFAEEKGLDFIS

ZjDFR2 SSSAVRIRDDAQPVLDETTWSSVQLCERMQL---WYALAKVYAEKAAWEFAKENDIDLVT

ZjDFR3 SMAAVAYNGKPRTVVDETWFSDPEICAKLQQ---WYVVSKTLAEEAAWKFARDNGFEIVT

ZjDFR1 IIPTLVVGPFLSTAMPPSLVTALALVTRNEPHYSILKQVQFVHLDDLCDAEIYLFEHPDA

ZmDFR IIPTLVVGPFISASMPPSLITALALITGNAPHYSILKQVQLIHLDDLCDAEIFLFENPAA

HvDFR IIPTLVVGPFLSAGMPPSLVTALALITGNEAHYSILKQVQLVHLDDLCDAMTFLFEHPEA

OsDFR VIPTLVVGPFISNGMPPSHVTALALLTGNEAHYSILKQVQFVHLDDLCDAEIFLFESPEA

AmDFR IIPPLVVGPFIMPTFPPSLITALSPITGNEAHYSIIKQCQYVHLDDLCEGHIFLFEYPKA

GhDFR IIPTLVVGPFITSTFPPSLVTALSLITGNEAHYSIIKQGQYVHLDDLCECHIYLYENPKA

VmDFR IIPTLVVGPFISPTFPPSLITVLSPITGNEAHYSIIKQCQYVHLDDLCKYLMFLLEHPEA

PhDFR IIPPLVVGPFITPTFPPSLITALSLITGNEAHYCIIKQGQYVHLDDLCEAHIFLYEHPKA

LjDFR2 IIPPLVVGSFLMPTMPPSLITALSPITGNEAHYSIIKQGQYVHLDDLCLAHIFLFEHPES

LjDFR3 IIPSLVVGSFLMPTLPPSLTTALSPITGNEAHYSIIKQGQYVHLDDLCLAHIFLFEHPKS

LjDFR5 VIPPLVVGPFLMPTMPPSLITALSLITGNEAHYSIIKQGQYVHLDDLCLAHIFLFENPKA

RhDFR IIPTLVIGPFLMPSMPPSLITGLSPLTGNESHYSIIKQGQFIHLDDLCQSHIYLYEHPKA

VvDFR IIPTLVVGPFIMSSMPPSLITALSPITGNEAHYSIIRQGQFVHLDDLCNAHIYLFENPKA

AtDFR IIPTLVVGPFITTSMPPSLITALSPITRNEAHYSIIRQGQYVHLDDLCNAHIFLYEQAAA

ZjDFR2 VLPSFVIGPSLSKELCVTASDVLGLLQGDTARFSSYGRMGYVHIDDVASSHILVYEALEA

ZjDFR3 INPAMVIGPLLQPTLNTSAEAILKLINGSSSTYSNVT-LGWVNVKDVALAHILAYEVPSA

ZjDFR1 AGRYVCSSDDATIHGLAAMLRERYPEYDIPESFPGIDDDLPPVHFSSKKLLDHGFRFRYT

ZmDFR AGRYVCSSHDVTIHGLAAMLRDRYPEYDVPQRFPGIQDDLQPVRFSSKKLQDLGFTFRYT

HvDFR NGRYICSSHDATIHGLARMLQDRFPEYDIPQKFAGVDDNLQPIHFSSKKLLDHGFSFRYT

OsDFR RGRYVCSSHDATIHGLATMLADMFPEYDVPRSFPGIDDHLQPVHFSSWKLLAHGFRFRYT

AmDFR EGRYICSSHDATIYDIAKLITENWPEYHIPDEFEGIDKDIPVVSFSSKKMIGMGFIFKYT

GhDFR KGRYICSSHDATIHQLAKIIKDKWPEYYIPTKFPGIDEELPIVSFSSKKLIDTGFEFKYN

VmDFR EGRYICSSHDATIYDLAKMMRRNGPGTMSPNEFKGIDKELPIMSFSSKKLLVIGFKFKYN

PhDFR DGRFICSSHHAIIYDVAKMVREKWPEYYVPTEFKGIDKDLPVVSFSSKKLTDMGFQFKYT

LjDFR2 EGRYICSASEATIHDIAKLINSKYPEYNIPTKFKNIPDELELVRFSSKKIKDMGFEFKYS

LjDFR3 EGRYICSASEATIHDIAKLINSKYPEYNVPTKFKNIPDELELVRFSSKKIKDMGFEFKYS

LjDFR5 QGRYMCSAYEATIHEVARMINKKYPEFNVPTKFKDIPDELDIIKFSSKKITDLGFKFKYS

RhDFR EGRYICSSHDATIHEIAKLLKGKYPEYNVPTTFKGIEENLPKVHFSSKKLLETGFEFKYS

VvDFR EGRYICSSHDCIILDLAKMLREKYPEYNIPTEFKGVDENLKSVCFSSKKLTDLGFEFKYS

AtDFR KGRYICSSHDATILTISKFLRPKYPEYNVPSTFEGVDENLKSIEFSSKKLTDMGFNFKYS

ZjDFR2 TGRYLCSSVVLDNNELVSLLAKRYPIFPIPRRLNN-PYGEQSYQLNTSKLQGLGFKFKG-

ZjDFR3 NGRYCIVERVLHYSDVVNVIRKMYPTIPLPDKCADDKFVP-TYQVSKEKIRSLGIELIP-

ZjDFR1 VQDMFDEAIRTCREKGLIP

ZmDFR LEDMFDAAIRTCQEKGLIP

HvDFR TEDMFDAAIHTCRDKGLIP

OsDFR LEDMFEAAVRTCREKGLLP

AmDFR LEDMVRGAIDTCREKGMLP

GhDFR LEDMFKGAIDTCREKGLLP

VmDFR LEDMFRGAIDTWQEKGLLP

PhDFR LEDMYKGAIDTCRQKQLLP

LjDFR2 LEDMYTGAIDTCKEKGLLP

LjDFR3 LEDMYTGAIDTCKEKGLLP

LjDFR5 LEDMYTGAVETCREKGLLP

RhDFR LEDMFVGAVDACKEKGLLP

VvDFR LEDMFTGAVDTCRAKGLLP

AtDFR LEEMFIESIETCRQKGFLP

ZjDFR2 VQEMFDDCVQSLKDQGHL-

ZjDFR3 LETSIKETIESLKEKGFVS

B. Alignment of ZjANS2 homologs

GmANS1 LANNASGQLEWEDYFFHLVFPEDKRDLSIWPKKPDDYIEVTSEYAKRLRGLATKMLEALS

MtANS LANNASGQLEWEDYFFHCIFPEDKRDLSIWPKTPADYTKVTSEYAKELRVLASKIMEVLS

AtANS LANNASGQLEWEDYFFHLAYPEEKRDLSIWPKTPSDYIEATSEYAKCLRLLATKVFKALS

MdANS LANNASGQLEWEDYFFHCVYPEDKRDLSIWPQTPADYIEATAEYAKQLRELATKVLKVLS

VvANS LANNASGQLEWEDYFFHLIFPEDKRDMTIWPKTPSDYVPATCEYSVKLRSLATKILSVLS

NtANS LANSACGQLEWEDYFFHCVFPEDKCNLSIWPKTPTDYIPATSEYAKQIRNLATKILAVLS

GhANS LANNASGQLEWEDYFFHLVFPEEKRDLTIWPTTPSDYTDATTEYAKQLRALATKILPALS

ZjANS1 LATNASGQREWEDYLFHLLHPDGLADHALWPAHPPDYVATTREFGRRVRELASRLLAILS

ZmANS LATNTCGQREWEDYLFHLVHPDGLADHALWPAYPPDYIAATRDFGRRTRDLASTLLAILS

OsANS LAANASGKREWEDYLFHLVHPDHLADHSLWPANPPEYVPVSRDFGGRVRTLASKLLAILS

TaANS LAGSAGGKREWEDYLFHMLHPDARADHARWPAHPPEYVPVTKAFGEHVSALSSRLLAILS

ZjANS2 LANDDS-VLDWRDYLDHHTLPESRRNPSHWPDFVPGYRDTVVKYSNSMKDLAQRLLRIIS

ZjANS3 LVKFEDQTLDWCDRLHLRVEPEAERNCSLWPKHPESFRALLHEYTLSCRRIRDCILQAMA

GmANS1 IGLGLEGGRLEKEVGGMEELLLQLKINYYPICPQPELALGVEAHTDVSSLTFLLHNMVPG

MtANS LELGLEGGRLEKEAGGMEELLLQMKINYYPICPQPELALGVEAHTDVSSLTFLLHNMVPG

AtANS VGLGLEPDRLEKEVGGLEELLLQMKINYYPKCPQPELALGVEAHTDVSALTFILHNMVPG

MdANS LGLGLDEGRLEKEVGGLEELLLQMKINYYPKCPQPELALGVEAHTDVSALTFILHNMVPG

VvANS LGLGLEEGRLEKEVGGMEELLLQKKINYYPKCPQPELALGVEAHTDVSALTFILHNMVPG

NtANS IGLRLEEGRLEKEVGGMEDLLLQMKINYYPKCPQPELALGVEAHTDVSALTFILHNMVPG

GhANS LGLGLEEGRLEKEVGGIEELILQLKINYYPKCPQPELALGVEAHTDVSALTFILHNMVPG

ZjANS1 LGLGLREHKLEDELTNQEDLLLQLKINYYPRCPQPELAVGVEAHTDVSALSFILHNGVPG

ZmANS MGLGTDGDALEKALTT--DLLLQLKINYYPRCPQPELAVGVEAHTDVSALSFILHNGVPG

OsANS LGLGLPEETLERRLRG--DLLLQLKINYYPRCPRPDLAVGVEAHTDVSALSFILHNGVPG

TaANS LGLGVPADTLERRLRL--DLLLKLKINYYPRCPQPELAVGVEAHTDVSALSVILTNGVPS

ZjANS2 ECLNLP-PSYIEEAVG--EVYQNITVSYYSPCPQPDLALGLQSHSDMGAITLLIQDDVGG

ZjANS3 KTLGLNEDYIISHFTD--KAPTFARFNYYPPCPRPDLVFGIKPHSDSGVLTILLVDDVAG

GmANS1 LQLFYQGQWFTAKCVPNSILMHIGDTIEILSNGKYKSILHRGLVNKEKVRISWAMFCEPP

MtANS LQLFYEGKWVTAKCVPDSILMHIGDTIEILSNGKYKSILHRGLVNKEKVRISWAVFCEPP

AtANS LQLFYEGKWVTAKCVPDSIVMHIGDTLEILSNGKYKSILHRGLVNKEKVRISWAVFCEPP

MdANS LQLFYEGKWVTAKCVPNSIVMHIGDTLEILSNGKYKSILHRGMVNKEKVRISWAVFCEPP

VvANS LQLFYEGKWVTAKCVPNSIIMHIGDTIEILSNGKYKSILHRGLVNKEKVRISWAVFCEPP

NtANS LQLFYEGQWVTAKCVPNSIIMHIGDTLEILSNGKYKSILHRGVVNKEKIRISWAIFCEPP

GhANS LQLFYDGQWVSAQCVPDSIILHIGDALEILSNGEYKSILHRGLVNKEKVRISWAVFCEPP

ZjANS1 LQVLHGGRWVTARSEPGTMIVHVGDALEILSNGRYTSVLHRGLVNREAVRVSWVVFCEPP

ZmANS LQVLHGARWVTARHEPGTIIVHVGDALEILSNGRYTSVLHRGLVNREAVRISWVVFCEPP

OsANS LQVHHAGSWVTARPEPGTIVVHVGDALEILTNGRYTSVLHRGLVSRDAVRLSWVVFCEPP

TaANS LQVLHPGNWVTARDEPGTLVVHVGDSLEILSNGRYTSVLHRGLVNRQAVRVSWVVFAQPP

ZjANS2 LEVMKDGMWIPVPPLPDGILVILADQTEIITNGRYRSSVHRAVVNAEHARLSVATFYDPS

ZjANS3 LQILRDDKWHNVPTSPHRLLVNLGDYSEIMSNGIFKSPVHRAVANMEKERISLAMFHGLD

GmANS1 KEKIILQPLPELVTETEPARFPPRTFAQHIHHKLFRK

MtANS KEKIILKPLPELVTEKEPARFPPRTFAQHIHHKLFRK

AtANS KDKIVLKPLPEMVSVESPAKFPPRTFAQHIEHKLFGK

MdANS KEKIILKPLPETVSEDEPAMFPPRTFAEHIQHKLFRK

VvANS KEKIILKPLPETVSETEPPLFPPRTFSQHIQHKLFRK

NtANS KEKIILKPLPETITEAEPPRFPPRTFAQHMAHKLFKK

GhANS KEKIVLKPLPETVSEAEPPLFPPRTFRQHMEHKLFRK

ZjANS1 PDAVLLRPLPELVTEEEPARFTPRTFKEHLDRKLFKK

ZmANS PDSVLLHPLPELVTEGHPARFTPRTFKQHLDRKLFKK

OsANS PESVLLQPVQELLADGGKPLFAPRTFKQHVQRKLFKK

TaANS PDSVLLGPLPELVQGYSRRRMINRTTRLRSRRKVVKK

ZjANS2 KSRKICT-APLLVSNDEPKKYRDIVYGDYVSS-WYSK

ZjANS3 PEKEIEPAVDCYMKSN----------QHGIGN-----
