# Supplementary material for: De Novo Transcriptome Analysis to Identify Anthocyanin Biosynthesis Genes Responsible for Tissue-Specific Pigmentation in Zoysiagrass (Zoysia japonica Steud.)
Source: PLoS One. 2015 Apr 23;10(4):e0124497. doi: 10.1371/journal.pone.0124497 (PMC4408010; doi:10.1371/journal.pone.0124497)
Supplement: S1 Table — (DOCX) [file pone.0124497.s021.docx]

**Table S1.** Summary of filtered and assembled RNA-seq data generated on Illumina HiSeq 2000 platform using RNA isolated from spike tissues of *Z. japonica* species.

|  | *Z. japonica*  'Anyang-jungji' | *Z. japonica* 'Greenzoa' |
| --- | --- | --- |
| Total read pairs sequenced | 22,228,361,386 | 22,724,700,838 |
| Number of reads obtained after quality filtering | 19,212,882,206 | 19,253,496,909 |
| Average read size used for assembly (bp) | 96.4 | 96.3 |
| Properly mapped read pairs (%) | 81.3 | 80.6 |
