# Supplementary material for: De Novo Transcriptome Analysis to Identify Anthocyanin Biosynthesis Genes Responsible for Tissue-Specific Pigmentation in Zoysiagrass (Zoysia japonica Steud.)
Source: PLoS One. 2015 Apr 23;10(4):e0124497. doi: 10.1371/journal.pone.0124497 (PMC4408010; doi:10.1371/journal.pone.0124497)
Supplement: S2 Table — (DOCX) [file pone.0124497.s022.docx]

**Table S2.** *De novo* transcriptome assembly details using multiple assemblers.

|  |  | Avg. contig size (bp) | Median contig  size (bp) | N50 contig size (bp) | Max. size (bp) | Min. size (bp) | Total contig number | Total length (bp) |
| --- | --- | --- | --- | --- | --- | --- | --- | --- |
| AJ | Trinity | 1,451.2 | 1,163 | 2,043 | 13,605 | 200 | 20,338 | 29,515,235 |
|  | Velvet | 1,242.5 | 1,015 | 1,643 | 15,279 | 200 | 12,758 | 15,851,569 |
|  | CLC | 986.6 | 714 | 1,420 | 15,206 | 200 | 25,850 | 25,502,199 |
|  | Merged | 982.4 | 699 | 1,395 | 15,205 | 200 | 28,561 | 28,057,682 |
| GZ | Trinity | 1,516.5 | 1,233 | 2,096 | 14,778 | 200 | 18,832 | 28,558,098 |
|  | Velvet | 1,252.2 | 1,010 | 1,668 | 13,073 | 200 | 12,691 | 15,891,637 |
|  | CLC | 989.8 | 706 | 1,438 | 15,359 | 200 | 26,445 | 26,174,934 |
|  | Merged | 984.9 | 715 | 1,415 | 15,359 | 200 | 28,984 | 28,547,216 |
