# Supplementary material for: De Novo Transcriptome Analysis to Identify Anthocyanin Biosynthesis Genes Responsible for Tissue-Specific Pigmentation in Zoysiagrass (Zoysia japonica Steud.)
Source: PLoS One. 2015 Apr 23;10(4):e0124497. doi: 10.1371/journal.pone.0124497 (PMC4408010; doi:10.1371/journal.pone.0124497)
Supplement: S4 Table — (DOCX) [file pone.0124497.s024.docx]

**Table S4.** NCBI accession number of ACTINs.

| Species | Accession number |
| --- | --- |
| *Arabidopsis thaliana* | NM_179953.2 |
| *Brassica rapa* | JN120480.1 |
| *Brachypodium distachyon* | XM_003560541.1 |
| *Chlamydomonas reinhardtii* | D50839.1 |
| *Glycine max* | NM_001253024.2 |
| *Hordeum vulgare* | AK251023.1 |
| *Medicago truncatula* | XM_003621971.1 |
| *Oryza sativa* | AB047313.1 |
| *Physcomitrella patens* | XM_001782636.1 |
| *Populus trichocarpa* | XM_006379531.1 |
| *Selaginella moellendorffii* | XM_002980705.1 |
| *Setaria italica* | XM_004981913.1 |
| *Solanum lycopersicum* | XM_004236699.1 |
| *Solanum tuberosum* | XM_006351284.1 |
| *Sorghum bicolor* | X79378.1 |
| *Vitis vinifera* | XM_002283554.2 |
| *Zea mays* | NM_001156990.1 |
| *Zoysia japonica* | GU290545.1 |
