# Supplementary material for: De Novo Transcriptome Analysis to Identify Anthocyanin Biosynthesis Genes Responsible for Tissue-Specific Pigmentation in Zoysiagrass (Zoysia japonica Steud.)
Source: PLoS One. 2015 Apr 23;10(4):e0124497. doi: 10.1371/journal.pone.0124497 (PMC4408010; doi:10.1371/journal.pone.0124497)
Supplement: S6 Table — (DOCX) [file pone.0124497.s026.docx]

**Table S6.** Zoysiagrass unigenes used for qRT-PCR analysis.

| Genes | REFSEQ Match | Accession No. | E-value | ID^a^ | Region of ID^b^ | % Cov^c^ | Amplicon region^d^ |
| --- | --- | --- | --- | --- | --- | --- | --- |
| *ZjPAL1* | *SiPAL* | XP_004953154.1 | 0 | 93 | *1-718* | 99 | 551-639 (1,884) |
| *ZjPAL2* | *ZmPAL* | NP_001241797.1 | 0 | 94 | *1-701* | 99 | 997-1,113 (2,106) |
| *ZjPAL3* | *SiPAL* | XP_004976238.1 | 0 | 87 | 230-702* | 99 | 576-681 (1,425) |
| *ZjCHS1* | *SiCHS* | XP_004979391.1 | 0 | 95 | *1-399* | 99 | 896-983 (1,206) |
| *ZjCHS2* | *ZmCHS* | NP_001149508.1 | 1.00E-127 | 84 | 178-412* | 98 | 263-355 (690) |
| *ZjCHI1* | *AtCHI* | NP_567140.1 | 5.00E-37 | 34 | 72-275* | 48 | 526-633 (1,284) |
| *ZjCHI2* | *ZmCHI* | NP_001149585.1 | 8.00E-88 | 84 | 112-274* | 99 | 232-338 (495) |
| *ZjCHI3* | *BdCHI* | XP_003571233.1 | 3.00E-100 | 77 | 68-265* | 99 | 139-256 (627) |
| *ZjCHI4* | *SiCHI* | XP_004981264.1 | 3.00E-100 | 84 | 54-233* | 98 | 160-261 (537) |
| *ZjF3H1* | *ZmF3H1* | NP_001105695.1 | 0 | 88 | *1-336* | 90 | 310-429 (1,116) |
| *ZjF3H2* | *SiF3H* | XP_004985921.1 | 0 | 89 | 55-336* | 98 | 22-141 (855) |
| *ZjF3’H* | *BdF3’H* | XP_003577475.1 | 0 | 77 | 21-529 | 96 | 1,287-1,383 (1,599) |
| *ZjF3’5’H* | *SiF3’5’H* | XP_004984278.1 | 1.00E-32 | 97 | 456-517 | 92 | 98-201 (201) |
| *ZjDFR1* | *SiDFR* | XP_004969260.1 | 0 | 86 | *1-365* | 96 | 578-716 (1,116) |
| *ZjDFR2* | *ZmDFR1* | NP_001105644.1 | 0 | 89 | *1-331* | 99 | 265-364 (990) |
| *ZjDFR3* | *BdDFR* | XP_003567727.1 | 0 | 83 | 10-329* | 97 | 144-262 (982) |
| *ZjANS1* | *ZmANS* | NP_001106074.1 | 5.00E-180 | 74 | *1-395* | 99 | 132-244 (1,179) |
| *ZjANS2* | *SiANS* | XP_004964770.1 | 1.00E-137 | 90 | 117-343* | 99 | 100-216 (685) |
| *ZjANS3* | *ZmANS* | NP_001152138.1 | 8.00E-142 | 63 | *1-314* | 95 | 379-496 (987) |
| *ZjUFGT1* | *SiUFGT1* | XP_004955859.1 | 6.00E-147 | 72 | 187-470* | 99 | 320-407 (855) |
| *ZjUFGT2* | *SiUFGT1* | XP_004955859.1 | 1.00E-143 | 70 | 187-470* | 99 | 321-408 (856) |
| *ZjFLS* | *SiFLS* | XP_004954034.1 | 0 | 79 | *3-333* | 98 | 736-823 (1,011) |
| *ZjMYB1* | *AtMYB-IF35* | NP_001105092.1 | 2.00E-67 | 94 | *1-109 | 99 | 164-276 (329) |
| *ZjMYB2* | *AtMYB4* | NP_195574.1 | 9.00E-68 | 98 | *1-105 | 69 | 297-402 (451) |

^a^Percentage sequence identity (ID), based on amino acid sequence.

^b^Asterisks at left and right of the region indicate the presence of predicted start and stop codons, respectively.

^c^Percentage coverage, the percentage of total predicted protein length present in unigene sequence.

^d^The nucleotide region within each unigene that qRT-PCRprimers were designed to amplify, with total unigene coding sequence length in parentheses.
