# Supplementary material for: De Novo Transcriptome Analysis to Identify Anthocyanin Biosynthesis Genes Responsible for Tissue-Specific Pigmentation in Zoysiagrass (Zoysia japonica Steud.)
Source: PLoS One. 2015 Apr 23;10(4):e0124497. doi: 10.1371/journal.pone.0124497 (PMC4408010; doi:10.1371/journal.pone.0124497)
Supplement: S7 Table — (DOCX) [file pone.0124497.s027.docx]

**Table S7.** FPKM value of anthocyanin biosynthesis related transcripts.

| Gene name | AJ transcript | GZ transcript | AJ FPKM^*^ | GZ FPKM^*^ |
| --- | --- | --- | --- | --- |
| *ZjPAL1* | aj_contig_963 | gz_contig_1100 | 120.11 | 106.28 |
| *ZjPAL2* | aj_contig_171 | gz_contig_514 | 551.02 | 514.24 |
| *ZjPAL3* | aj_contig_93 | gz_contig_334 | 255.84 | 132.20 |
| *ZjCHS1* | aj_contig_1774 | gz_contig_821 | 255.10 | 674.06 |
| *ZjCHS2* | aj_contig_28560 | gz_contig_3068 | 2.80 | 117.62 |
| *ZjCHI1* | aj_contig_17328 | gz_contig_11760 | 11.01 | 14.02 |
| *ZjCHI2* | aj_contig_20572 | gz_contig_5945 | 10.25 | 20.68 |
| *ZjCHI3* | aj_contig_20378 | gz_contig_12665 | 6.19 | 10.68 |
| *ZjCHI4* | aj_contig_22952 | gz_contig_12577 | 36.57 | 54.30 |
| *ZjF3H1* | aj_contig_11511 | gz_contig_2786 | 99.24 | 367.83 |
| *ZjF3H2* | aj_contig_12276 | | 26.01 | 5.09 |
| *ZjDFR1* | aj_contig_14208 | | 78.95 | 1.12 |
| *ZjDFR2* | aj_contig_28561 | gz_contig_117 | 1.73 | 122.93 |
| *ZjDFR3* | aj_contig_2500 | gz_contig_6946 | 153.48 | 96.34 |
| *ZjANS1* | aj_contig_7856 |  | 72.92 | 0.29 |
| *ZjANS2* | aj_contig_898 | gz_contig_19130 | 14.30 | 15.52 |
| *ZjANS3* | aj_contig_6803 | gz_contig_4405 | 13.96 | 17.99 |
| *ZjFLS* | aj_contig_14235 | gz_contig_2392 | 35.69 | 361.12 |
| *ZjUFGT1* | aj_contig_9283 | gz_contig_4733 | 7.95 | 16.06 |
| *ZjUFGT2* | aj_contig_9283 | gz_contig_4735 | 6.72 | 11.22 |
| *ZjF3'H* | aj_contig_8021 | gz_contig_5629 | 33.48 | 80.64 |
| *ZjF3'5'H* | aj_contig_24141 | gz_contig_24420 | 8.20 | 14.28 |
| *ZjMYB1* | aj_contig_13033 | gz_contig_11866 | 15.94 | 38.77 |
| *ZjMYB2* | aj_contig_13209 | gz_contig_7425 | 19.57 | 9.27 |
| * FPKM : Fragments Per Kilobase of exon per Million fragments mapped | | | | |
