# Supplementary material for: De Novo Transcriptome Analysis to Identify Anthocyanin Biosynthesis Genes Responsible for Tissue-Specific Pigmentation in Zoysiagrass (Zoysia japonica Steud.)
Source: PLoS One. 2015 Apr 23;10(4):e0124497. doi: 10.1371/journal.pone.0124497 (PMC4408010; doi:10.1371/journal.pone.0124497)
Supplement: S8 Table — (DOCX) [file pone.0124497.s028.docx]

**Table S8.** Accession number of DFR and ANS homologous proteins.

| Gene product^a^ | Accession number |
| --- | --- |
| AmDFR | P14721.1 |
| AtDFR | NP_199094.1 |
| GhDFR | P51105.1 |
| HvDFR | P51106.1 |
| LjDFR2 | BAE19949.1 |
| LjDFR3 | BAE19950.1 |
| LjDFR5 | BAE19953.1 |
| OsDFR | BAA36183.1 |
| PhDFR | P14720.2 |
| RhDFR | BAA12723.1 |
| VmDFR | AAL89714.1 |
| VvDFR | AAX12423.1 |
| ZmDFR | NP_001152467.1 |
| AtANS | NP_194019.1 |
| GhANS | AAY15744.1 |
| GtANS | BAE44202.1 |
| MdANS | P51091.1 |
| NtANS | BAM37963.1 |
| OsANS | CAA69252.1 |
| PcANS | ABB70119.1 |
| TaANS | BAE98276.1 |
| VvANS | NP_001268147.1 |
| ZmANS | P41213.1 |

^a^Am, *Antirrhinum majus*, At, *Arabidopsis thaliana*; Gh, *Gerbera hybrid*; Gt, *Gentiana triflora*; Hv, *Hordeum vulgare*; Lj, *Lotus japonicus*; Md, *Malus domestica*; Nt, *Nicotiana tabacum*; Os, *Oryza saiva*; Pc, *Pyrus communis*; Ph, *Petunia hybrid*; Rh, *Rosa hybrid*; Ta, *Triticum aestivum*; Vm, *Vaccinium macrocarpon*; Vv, *Vitis vinifera*; Zm, *Zea mays*.
