# Supplementary material for: De Novo Transcriptome Analysis to Identify Anthocyanin Biosynthesis Genes Responsible for Tissue-Specific Pigmentation in Zoysiagrass (Zoysia japonica Steud.)
Source: PLoS One. 2015 Apr 23;10(4):e0124497. doi: 10.1371/journal.pone.0124497 (PMC4408010; doi:10.1371/journal.pone.0124497)
Supplement: S9 Table — (DOCX) [file pone.0124497.s029.docx]

**Table S9.** Characteristics of *Zoysia* species.

| Cultivar | Species | Leaf color | Stolon color |
| --- | --- | --- | --- |
| AJ | *Z. japonica* | green | purple |
| Meyer | *Z. japonica* | green | purple |
| Senock | *Z. sinica* x *Z. matrella* | green | purple |
| Yaji | *Z. japonica* | green | purple |
| Gumjandi | *Z. matrella* | green | purple |
| Konhee | *Z. matrella* | green | purple |
| GZ | *Z. japonica* | green | green |
| Millock | Zoysia cultivar | green | green |
| Zenith | *Z. japonica* | green | green |
