# Supplementary material for: De Novo Transcriptome Analysis to Identify Anthocyanin Biosynthesis Genes Responsible for Tissue-Specific Pigmentation in Zoysiagrass (Zoysia japonica Steud.)
Source: PLoS One. 2015 Apr 23;10(4):e0124497. doi: 10.1371/journal.pone.0124497 (PMC4408010; doi:10.1371/journal.pone.0124497)
Supplement: S10 Table — (DOCX) [file pone.0124497.s030.docx]

**Table S10.** Primer sequences used in this study.

| Genes | Sequences | Purpose |
| --- | --- | --- |
| ZjPAL1-F | CGAATGTTATTGCCGTCCTT | qRT-PCR |
| ZjPAL1-R | GGTGAGGTGGTCGGTGTACT | qRT-PCR |
| ZjPAL2-F | CCTAAGCAGGACAGGTACGC | qRT-PCR |
| ZjPAL2-R | GTTGTCGTTGACGGAGTTGA | qRT-PCR |
| ZjPAL3-F | CTACAACAACGGGCTGACCT | qRT-PCR |
| ZjPAL3-R | GAGCTCGGAGCAGTAGGATG | qRT-PCR |
| ZjCHS1-F | CCGACTGGAACTCCATCTTC | qRT-PCR |
| ZjCHS1-R | TCCTTGTCGAGCTTGACCTT | qRT-PCR |
| ZjCHS2-F | CAGAGGAGGGCATCAAGTTC | qRT-PCR |
| ZjCHS2-R | GCTCCTTCATCAGCTTCCTG | qRT-PCR |
| ZjCHI1-F | AGGATGCTTGCTGACTCCAT | qRT-PCR |
| ZjCHI1-R | AGCAACACAAGCACAACCTG | qRT-PCR |
| ZjCHI2-F | GAACAGGAGGACACCAAAGG | qRT-PCR |
| ZjCHI2-R | TGGTTTGGTTCCCTCAAAAG | qRT-PCR |
| ZjCHI3-F | GCTGCTGCATTCTATGTGGA | qRT-PCR |
| ZjCHI3-R | CCGGTGCTTTAAAAATGGAG | qRT-PCR |
| ZjCHI4-F | GCCGAGAAGGTGACTGAGAA | qRT-PCR |
| ZjCHI4-R | CTTGAACGCCTCCTTGAACT | qRT-PCR |
| ZjF3H1-F | ATGTCGAACCGGAGCTTATC | qRT-PCR |
| ZjF3H1-R | CACGGGGTACGAGAAGTAGG | qRT-PCR |
| ZjF3H2-F | GGATTCTTCCAGGTGCTGAA | qRT-PCR |
| ZjF3H2-R | GTCGTCGGAGTAGAGCTTGG | qRT-PCR |
| ZjF3’H-F | CCCACTAGAGTTCCGACCAG | qRT-PCR |
| ZjF3’H-R | CGCACCAAACGGAATAAGAT | qRT-PCR |
| ZjF3’5’H-F | ATGTCCAGCTTCTCCTCGC | qRT-PCR |
| ZjF3’5’H-R | TCAGGCCGCAGCGTAG | qRT-PCR |
| ZjDFR1-F | GCCTGGACCTTATCAGCATC | qRT-PCR |
| ZjDFR1-R | AACTGCACCTGCTTGAGGAT | qRT-PCR |
| ZjDFR2-F | GTCCTCGCTAACTGCGATTC | qRT-PCR |
| ZjDFR2-R | GAAACGGGTTCTTCTTGCAC | qRT-PCR |
| ZjDFR3-F | TGGATGGAGCAAAGGATAGG | qRT-PCR |
| ZjDFR3-R | GTGATAGAAGGGCGAAGCAG | qRT-PCR |
| ZjANS1-F | CTTCGACCTTTTGGCTGAAC | qRT-PCR |
| ZjANS1-R | ACTGCGGCTGATCCTTCTT | qRT-PCR |
| ZjANS2-F | GGATTTCGTCCCTGGTTACA | qRT-PCR |
| ZjANS2-R | ATATAAGAGGGCGGCAGGTT | qRT-PCR |
| ZjANS3-F | GACCTGGTGAAATTCGAGGA | qRT-PCR |
| ZjANS3-R | CTCTGAACGATTCGGGATGT | qRT-PCR |
| ZjUFGT1-F | GCTTGGCATCTATGGAGGAG | qRT-PCR |
| ZjUFGT1-R | TGTCGAATCACCCACAGAAA | qRT-PCR |
| ZjUFGT2-F | GCTTGGCATCTATAGAGCAG | qRT-PCR |
| ZjUFGT2-R | TGTCGGATTACCCACAAAAA | qRT-PCR |
| ZjFLS-F | CACTGGTACGACGCCAAGTA | qRT-PCR |
| ZjFLS-R | CCTTGTACTCCCCGTTGCT | qRT-PCR |
| ZjMYB1-F | TGCGCTGGATCAACTATCTG | qRT-PCR |
| ZjMYB1-R | GATCAAGGACCACCTGTTGC | qRT-PCR |
| ZjMYB2-F | CTGCGGTGGATCAACTACCT | qRT-PCR |
| ZjMYB2-R | ACCATTTGTTGCCGACTAGG | qRT-PCR |
| DFR_5’RACE_GSP1 | CGTCGGCTTGATCACCTCGTTCTC | RACE |
| DFR_5’RACE_GSP2 | CGTCGGCTTGATCACCTCGTTCTC | RACE |
| DFR_3’RACE_GSP1 | GGCCTGGACCTTATCAGCATCATCC | RACE |
| DFR_3’RACE_GSP2 | GAACGAGCCGCACTACTCGATCCTC | RACE |
| ANS_5’RACE_GSP1 | GCACTGCGGCTGATCCTTCTTGTC | RACE |
| ANS_5’RACE_GSP2 | GGGGTGCAGGAGGTGGAAGAGGTA | RACE |
| DFR_Tail_R1 | CGTCGGCTTGATCACCTCGTTCTC | TAIL-PCR |
| DFR_Tail_R2 | CGTCGGCTTGATCACCTCGTTCTC | TAIL-PCR |
| DFR_Tail_R3 | GATGGACAGCCGCTCCTTCGCT | TAIL-PCR |
| ANS_Tail_R1 | CAGCCAAAAGGTCGAAGGCGTCG | TAIL-PCR |
| ANS_Tail_R2 | GTCGGCGGGTCGGACGTACTC | TAIL-PCR |
| ANS_Tail_R3 | GCTGCAGCACCGTCGAAGATG | TAIL-PCR |
| ZjDFR1_BS_F | GAYGGGTGAGATTYATTTYGTG | BS-seq |
| ZjDFR1_BS_R | CTTCACCTCCATCGCCTCTTC | BS-seq |
| ZjANS1_BS_F | GGYGYTAAGGTATYTTGAGGGTGATG | BS-seq |
| ZjANS1_BS_R | CACCTCCACACATACAACCACC | BS-seq |
| ZjDFR1_pLM302_F | GTCGACATGGGGGAGGTGGT | Protein expression |
| ZjDFR1_pLM302_R | AAGCTTTCACACATGCTCCCTTC | Protein expression |
| ZjANS1_pLM302_F | GAATTCGATGTCATCTTCGACGG | Protein expression |
| ZjANS1_pLM302_R | AAGCTTTCAGTTGGTTTTCGGTG | Protein expression |
